# Evaluating the potential cost-effectiveness of microarray patches to expand access to hepatitis B birth dose vaccination in low-and middle-income countries: A modelling study

**DOI:** 10.1371/journal.pgph.0000394

**Published:** 2022-05-23

**Authors:** Christopher P. Seaman, Mercy Mvundura, Collrane Frivold, Christopher Morgan, Courtney Jarrahian, Jess Howell, Margaret Hellard, Nick Scott

**Affiliations:** 1 Burnet Institute, Melbourne, Australia; 2 Department of Epidemiology and Preventive Medicine, Monash University, Melbourne, Australia; 3 PATH, Seattle, Washington, United States of America; 4 Jhpiego, The Johns Hopkins University Affiliate, Baltimore, Maryland, United States of America; 5 School of Population and Global Health, University of Melbourne, Melbourne, Australia; 6 Department of Gastroenterology, St Vincent’s Hospital, Melbourne, Australia; 7 Department of Medicine, University of Melbourne, Melbourne, Australia; 8 Doherty Institute and School of Population and Global Health, University of Melbourne, Melbourne, Australia; 9 Department of Infectious Diseases, The Alfred Hospital, Melbourne, Australia; Conservatoire National des Arts et Metiers, FRANCE

## Abstract

Timely birth dose vaccination is key for achieving elimination of hepatitis B, however, programmatic requirements for delivering current vaccine presentations to births outside of health facilities inhibits coverage within many low-and middle-income countries (LMICs). Vaccine technologies in development such as microarray patches (MAPs) could assist in overcoming these barriers, but procurement could incur higher per-dose commodity costs than current ten-dose (US$0.34) and single-dose (US$0.62) vial presentations, necessitating an evaluation of the economic value proposition for MAPs. Within 80 LMICs offering universal hepatitis B birth dose vaccination, the cost-effectiveness of using MAPs to expand coverage was evaluated using a mathematical model. We considered three potential per dose MAP prices (US$1.65, US$3.30, and US$5.00), and two potential MAP use-cases: (1) MAPs are used by lay-health workers to expand birth dose coverage outside of health facility settings, and (2) MAPs are also preferred by qualified health workers, replacing a proportion of existing coverage from vaccine vials. Analysis took the health system perspective, was costed in 2020 US$, and discounted at 3% annually. Across minimal (1% additional coverage) and maximal (10% additional and 10% replacement coverage) MAP usage scenarios, between 2.5 (interquartile range [IQR]: 1.9, 3.1) and 38 (IQR: 28,44) thousand DALYs were averted over the estimated 2020 birth cohort lifetime in 80 LMICs. Efficiency of MAPs was greatest when used to provide additional coverage (scenario 1), on average saving US$88.65 ($15.44, $171.22) per DALY averted at a price of US$5.00 per MAP. Efficiency was reduced when used to replace existing coverage (scenario 2); however, at prices up to US$5.00 per MAP, we estimate this use-case could remain cost-effective in at least 73 (91%) modelled LMICs. Our findings suggest even at higher procurement costs, MAPs are likely to represent a highly cost-effective or cost-saving mechanism to expand reach of birth dose vaccination in LMICs.

## Introduction

Timely birth dose vaccination is a key intervention for redressing ongoing global hepatitis B burden. Transmission of hepatitis B from mother-to-child at birth carries an up to 90% risk of progressing to chronic hepatitis B, the highest risk of any life point, and may be associated with higher rates of severe hepatic morbidity [1, 2]. Monovalent hepatitis B vaccination within the first 24-hours of life (timely birth dose) has been universally recommended for all newborns by the World Health Organisation (WHO) since 2009, as it provides highly efficacious prophylaxis against perinatal infection [3, 4]. Currently, this is provided in 80 low-and middle-income countries (LMICs) [5]; however, timely coverage of births outside of health facilities can remain a challenge due to programmatic requirements associated with current vaccine presentations [6, 7].

Delivery of a hepatitis B birth dose vaccine currently requires storage of vaccine vials in the standard cold chain (2–8°C) to the point of vaccine administration, leading to several potential coverage barriers. First, despite availability of a more suitable single-dose vial (SDV) paediatric monovalent hepatitis B vaccine; multi-dose vials (MDVs) are more frequently procured by LMICs due to lower per dose costs [8, 9]. A fear of vaccine wastage has been associated with health workers not opening MDVs, meaning missed vaccination opportunities [10], and although open MDVs can be stored for 28-days in a cold chain according to WHO policy [11], this duration may remain insufficient in some settings with low birth rates. Second, vaccines in vials require a needle and syringe for administration, and injectable formulations often require delivery by qualified health workers (e.g., a doctor, nurse, or midwife). For births outside of a health facility in LMICs, attendance of a qualified health worker is often delayed or absent [12], restricting timeliness and coverage of the birth dose. Lastly, within many LMICs, cold chain capacity for maintenance storage up to the point of birth is heavily constrained; with up to an estimated 75% of vaccination centres lacking adequate or sufficient cold chain capabilities [13].

Several innovations have previously been explored to overcome barriers to expanding birth dose coverage in LMICs, including a controlled temperature chain (CTC) approach and the use of compact prefilled auto-disable devices (CPADs) [14, 15]. A CTC approach leverages vaccine thermostability, facilitating storage of vaccines at temperatures beyond the standard cold chain. While no commercially available hepatitis B vaccines are licenced for the CTC approach, evidence supports storage of currently available vaccines at 37°C for up to four weeks with no loss of effectiveness [16]. While considered a priority vaccine for the CTC approach [17], and interest exists towards availability by national stakeholders [10, 18], sustained scalability of this approach is yet to be demonstrated within an immunisation program for other CTC vaccines. CPADs have been demonstrated as a feasible and acceptable means to increase timely HepB-BD coverage for births outside of health facilities [19–21]. However, despite licensure for over two decades, CPAD availability is constrained, and the evidence of integration into birth dose vaccination programs is limited to two countries (Indonesia and Timor-Leste) [22]. Further, CPADs require greater per-dose cold chain volumes compared to MDV presentations [23], potentially straining vaccine supply chains.

Although not yet commercially available, hepatitis B microarray patches (MAPs) are currently in clinical development with successful trials recently completed in human subjects (unpublished data, personal communication). This vaccine presentation could assist in overcoming barriers to increasing timely hepatitis B birth dose coverage in LMICs. MAPs eliminate the need for a needle and syringe to deliver a vaccine [24, 25]; instead, dry antigen is released from micron-scale projections which only penetrate the outermost layer of skin (stratum corneum) [26], and could facilitate vaccination of newborns by individuals with no or minimal prior training [27]. Acceptability of lay-health workers (such as trained community members) to deliver vaccines to infants has previously been established for CPADs [28], and evidence for MAPs indicates use in this capacity may also be considered acceptable [29–31]. Further, evidence on hepatitis B vaccine MAPs to date suggest they are likely to have a more robust thermostability profile than liquid equivalents, remaining potent after six-months exposure to 45°C [32], and eliciting similar or superior immune responses [32–35]. While more evidence is needed, this could enable MAPs to be used under a CTC or potentially entirely bypass the standard cold chain.

Despite MAPs favourable product profile for facilitating expanded hepatitis B birth dose coverage in LMICs, it is likely procurement costs will be higher than currently available liquid (vial) vaccines. Therefore, an economic evaluation prior to their commercial availability is needed to inform decision makers of their costs and benefits relative to alternate vaccine presentations. Previous evaluations for MAPs have shown use could provide a cost-effective means to improve both seasonal influenza and measles-rubella coverage [36–38]; but evidence is required for expanding hepatitis B birth dose coverage to currently unreached births in LMICs.

Using a mathematical model of hepatitis B transmission and progression, we aimed to investigate potential health and cost outcomes when MAPs are used to expand timely hepatitis B birth dose coverage in LMICs already providing a universal birth dose. Analysis focused on the incremental benefits MAPs could provide beyond existing vaccine administration approaches. Across a range of LMICs and global settings, we evaluate outcomes for several potential MAP introductory price points and assessed determinants of cost-effectiveness when compared to current vial presentations.

## Methods

### Model description

Mother-to-child hepatitis B transmission and subsequent disease progression were modelled using a previously validated mathematical model (Fig A in S1 Text) [14, 15]. Code and data to reproduce all reported analyses are available at https://github.com/ChrisSeaman-Burnet/HepB-BD-MAPs-Model.

A decision tree model was used to simulate mother-to-child transmission in a 2020 birth cohort for each modelled LMIC and WHO global region. Births could occur in either a facility (e.g., hospital, birthing clinic) or the community (e.g., home), with mother-to-child transmission risk a function of hepatitis B surface antigen (HBsAg) prevalence, maternal envelope antigen (HBeAg) prevalence and birth dose vaccination coverage. Effectiveness of birth dose vaccination decreased as time from birth increased (Table 1), with vaccines administered in one of four postpartum time-strata (day 1, day 2, days 3–7, days 8–41) [4, 15]. Hepatitis B prevalence was assumed constant for facility and community births; however, timeliness and coverage of existing birth dose coverage varied (Appendix 2 in S1 Text). We assumed facility births to have both a higher probability of vaccination, and more timely coverage, compared to community births [14, 39].

**Table 1:** Global model inputs.

Hepatitis B birth dose vaccine effectiveness (%) [4, 15]	Values (Uncertainty)	Notes
Timely administration (≤ 24-hours postpartum; Day 1)	95.3 (95% CI: 93.5, 96.7)	Uncertainty estimated using exact binomial methods from point estimate provided in source document, assuming α = 0.05
Day 2	88.9 (95% CI: 83.0, 92.8)	
Days 3–7	82.5 (95% CI: 70.7, 90.2)	
Days 8–41	52.3 (95% CI: 22.3, 80.7)	
**Hepatitis B disease progression (annual probability; %)** [1, 40, 41]		
Acute to Chronic^^^	88.5 (Range: 84.0, 93.0)	Only applied within first six-months of life; time-dependent after this point
Chronic to Immune	1.1 (Range: 0.1, 2.2)	
Chronic to Compensated Cirrhosis	1.9 (Range: 1.0, 2.4)	
Chronic to Hepatocellular Carcinoma	0.6 (Range: 0.3, 1.0)	
Compensated Cirrhosis to Decompensated Cirrhosis	3.9 (Range: 3.2, 4.6)	
Compensated Cirrhosis to Hepatocellular Carcinoma	4.8 (Range: 3.0, 6.6)	
Decompensated Cirrhosis to Hepatocellular Carcinoma	7.1 (Range: 4.8, 10.0)	Uncertainty estimated using exact binomial methods from point estimate provided in source document, assuming α = 0.05
**Hepatitis B attributable mortality (annual probability; %)** [42]		
Acute	0.07	Only point estimate available, ±5% error used for modelling uncertainty; estimated as mortality from fulminant hepatitis caused by perinatal transmission [43].
Decompensated Cirrhosis	16.2 (Range: 9.9, 20.0)	
Hepatocellular Carcinoma	54.5 (Range: 8.1, 60.5)	
**Disability weights associated with each disease state (annual)** [42]		
Chronic	0.011 (95% CI: 0.005, 0.021)	Proxied as “Abdominopelvic problem: mild”“ [44, 45]
Compensated Cirrhosis	0.123 (95% CI: 0.070, 0.176)	Drawn from lower bound of “Decompensated cirrhosis of the liver (without anaemia)” [45, 46]
Decompensated Cirrhosis	0.178 (95% CI: 0.123, 0.250)	“Decompensated cirrhosis of the liver (without anaemia)”
Hepatocellular Carcinoma	0.288 (95% CI: 0.193, 0.399)	“Diagnosis and primary therapy phase of liver cancer due to hepatitis B”
**Baseline Vaccine Commodity Costs (2020 US$)** [8, 47]		
Ten-/multidose vial (MDV)	0.34 (Range: 0.31, 0.41)	Includes cost of 0.5mL autodisable syringe, wastage, and disposal
Single dose vial (SDV)	0.62 (Range: 0.54, 0.71)	Includes cost of 0.5mL autodisable syringe, wastage, and disposal
MDV: SDV procurement ratio	3:1	

95%CI: 95% confidence interval, Range: Minimum value to maximum value

Subsequent progression of vertically acquired hepatitis B was simulated using a Markov model. All infections were assumed latent for a period of three weeks, prior to becoming acute. Acute infections lasted an average six-months, before approximately 90% progressed to chronic hepatitis B [1]. Within the model, chronic infections could: advance to compensated cirrhosis, decompensated cirrhosis, or hepatocellular carcinoma; persist, or spontaneously clear. Outcomes of infection were simulated over a lifetime horizon, with age-specific, all-cause mortality occurring in all model compartments and hepatitis B attributable mortality in acute, decompensated cirrhosis and hepatocellular carcinoma compartments.

### Inputs: Demography and epidemiology

HBsAg prevalence was taken from the WHO hepatitis B dashboard, reflective of population prevalence in 2019 [48]. HBeAg prevalence among HBsAg positive pregnant women was taken from a global review by Ott and colleagues, available in sex-specific ten-year age bands for Global Burden of Disease (GBD) regions [49]. For each modelled LMIC, prevalence estimates in relevant GBD regions (Table A in S1 Text) were weighted by the age distribution of new mothers between 2015–2020 [50], and uncertainty took the minimum and maximum values across the entire 15–49-year-old female age-range (reproductive age females). Presence of HBeAg increased risk of mother-to-child transmission from 17.5% to 80% for all LMICs, except those in sub-Saharan Africa where evidence suggests a reduced risk of vertical hepatitis B transmission [51, 52].

To ensure we only evaluated the impact of using MAPs to expand the reach of an existing hepatitis B birth dose program beyond traditional vaccine administration approaches using vials, the analysis was constrained to LMICs already offering a universal birth dose. WHO-UNICEF Estimates of National Immunization Coverage (WUENIC) data indicated 80 LMICs were providing the birth dose in 2019–2020, ranging between 21–99% coverage rate across countries (Table 2) [5]. Each LMIC also had a corresponding coverage rate estimate of the three dose infant series.

**Table 2:** Regional level model inputs.

	AFR	AMR	EMR	EUR	SEAR	WPR	All LMICs
LMICs included (n =)	8	20	9	17	8	18	80
**Demographics** [50, 53]							
Births (2020)	3,810,658	9,497,473	7,974,585	4,333,347	31,960,247	22,563,760	80,140,071
Life Expectancy, years (Range across included LMICs)	66.0 (57.2, 76.6)	75.4 (69.7, 80.0)	71.9 (64.3, 78.8)	73.9 (68.0, 78.4)	69.7 (66.8, 78.5)	75.6 (64.2, 76.6)	72.3 (57.2, 80.0)
Births in facilities, % (Range across included LMICs)	83.8 (69.8, 96.8)	95.8 (65.0, 99.9)	84.3 (56.3, 99.9)	97.6 (88.2, 99.9)	78.2 (37.1, 98.6)	96.1 (57.4, 99.9)	87.3 (37.1, 99.9)
**Epidemiology** [48, 49, 51, 52]							
2019 HBsAg prevalence, % (95% CI)	6.5 (5.8, 7.4)	0.8 (0.6, 1.2)	1.5 (1.2, 1.9)	3.5 (2.8, 4.9)	2.7 (2.3, 3.4)	6.0 (5.4, 6.6)	3.5 (3.1, 4.2)
HBeAg prevalence amongst HBsAg positive pregnant women, % (Range)	27.7 (15.4, 39.8)	31.2 (16.7, 43.3)	26.3 (15.0, 37.6)	28.8 (15.9, 41.3)	28.2 (15.6, 38.5)	33.3 (18.3, 48.8)	29.8 (16.4, 42.1)
HBeAg+ transmission risk, % (Range)	50.2 (24.9, 78.8)	80.0 (70.0, 90.0)	79.9 (69.8, 90.0)	80.0 (70.0, 90.0)	80.0 (70.0, 90.0)	80.0 (70.0, 90.0)	78.6 (67.8, 89.5)
HBeAg- transmission risk, % (Range)	8.4 (1.5, 18.0)	17.5 (5.0, 30.0)	17.5 (5.0, 30.0)	17.5 (5.0, 30.0)	17.5 (5.0, 30.0)	17.5 (5.0, 30.0)	17.1 (4.8, 29.4)
**Vaccination Coverage** [5]							
Hepatitis B birth dose coverage (HepB-BD), % (Range across included LMICs)	77.6 (21, 99)	63.5 (48, 99)	72.2 (38, 99)	94.8 (69, 99)	57.6 (21, 99)	88.9 (24, 99)	71.5 (21, 99)
Hepatitis B infant series coverage (HBV3), % (Range across included LMICs)	84.9 (72, 95)	77.4 (51, 99)	88.3 (70, 99)	92.5 (79, 99)	84.1 (77, 99)	94.9 (39, 99)	87.3 (39, 99)
**Annual Disease Management Costs, 2020 US$ (95% CI)** [44, 54, 55]							
Diagnosis*	4.60 (0.94, 10.85)	11.56 (2.12, 28.38)	6.66 (1.30, 16.12)	10.44 (1.94, 25.57)	4.19 (0.87, 9.97)	7.48 (1.47, 17.98)	6.56 (1.29, 15.82)
Chronic	26.93 (8.00, 119.83)	33.88 (9.18, 137.37)	28.99 (8.36, 125.10)	32.77 (9.00, 134.55)	26.52 (7.93, 118.95)	29.80 (8.53, 126.97)	28.88 (8.35, 124.80)
Compensated Cirrhosis	62.78 (39.59, 161.42)	95.36 (50.33, 226.04)	74.09 (43.30, 183.33)	93.24 (49.47, 221.69)	60.98 (39.17, 162.33)	75.42 (43.90, 184.47)	72.04 (42.74, 178.99)
Decompensated Cirrhosis	66.23 (40.82, 167.86)	112.15 (56.54, 256.98)	82.36 (46.26, 198.42)	109.51 (55.33, 251.84)	63.71 (40.22, 162.33)	83.96 (47.10, 199.19)	79.29 (45.44, 191.98)
Hepatocellular Carcinoma	64.65 (40.25, 164.90)	104.44 (53.69, 242.77)	78.56 (44.91, 191.49)	102.04 (52.64, 237.99)	62.46 (39.74, 160.11)	80.04 (45.62, 192.43)	75.96 (44.20, 186.02)

95%CI = 95% confidence interval, Range = Minimum value to maximum value.

*Diagnosis included as a one-time cost, incurred for each chronic hepatitis B infection resultant from mother-to-child transmission within the model.

*Abbreviations*: AFR: African WHO Region, AMR: American WHO Region, EMR: Eastern Mediterranean WHO Region, EUR: European WHO Region, HBsAg: Hepatitis B surface antigen, HBeAg: Hepatitis B envelope antigen, LMIC: Low-and middle-income country, SEAR: Southeast Asian WHO Region, WHO: World Health Organization, WPR: Western Pacific WHO Region

Data for individual LMICs available in the S1 File.

Annual probabilities of hepatitis B progression and non-acute hepatitis B mortality were taken from reviews and assumed as average across the lifetime [40, 41]. Acute mortality from vertical transmission was approximated as the rate of fulminant mortality, conditional upon perinatal transmission [43]. Age-specific all-cause mortality rates were calculated from life tables in the 2019 UN Population Prospects, and reflected expected mortality trends among a 2015–2020 birth cohort [50].

Analysis for the six WHO regions and an aggregate of all 80 LMICs (shown in tables as “all LMICs”) used parameter averages, population weighted by 2020 births, across relevant subsets of LMICs providing universal HepB-BD vaccinations and excluded those with missing data. For analysis across the 80 individual LMICs, missing data were imputed using WHO regional averages. Parameter values for each LMIC are available within a (S1 File).

### Inputs: Cost and health utilities

Presented costs are in 2020 United States Dollars (US$), adjusted using the consumer price index method, with resources valued at economic costs. Analysis took the health-system perspective, and future outcomes (health and economic) were discounted at 3% per annum.

### Vaccine modalities

Within the model, vaccine costing accounted for combinations of birth location (facility or community), vaccine presentation (vial or MAP) and health worker cadre (qualified health worker or lay health worker). For the baseline scenario, we assumed all existing birth dose vaccination coverage was from vaccine vials (combination of MDV and SDV) stored in the cold chain and administered by qualified health workers. Within evaluated scenarios, MAPs could be used by both qualified health workers and lay health workers. Expansion of coverage (i.e., reaching previously unvaccinated births) in the community was assumed to be delivered by lay health workers who reside in those communities; consistent with MAP product targets which specify use should require minimal or no prior training [27]. However, any use of MAPs by a qualified health worker was assumed to be attributable to a worker preference (e.g., motivated by enhanced simplicity and availability)–consistent with occurrences in trials employing CPADs to allow lay health workers to administer vaccinations [21]- and would only serve to replace existing coverage in both facility and community settings. MAPs were assumed to generate a non-inferior immune response compared to the vial presentation.

### Vaccination costs

**Fig 1:**
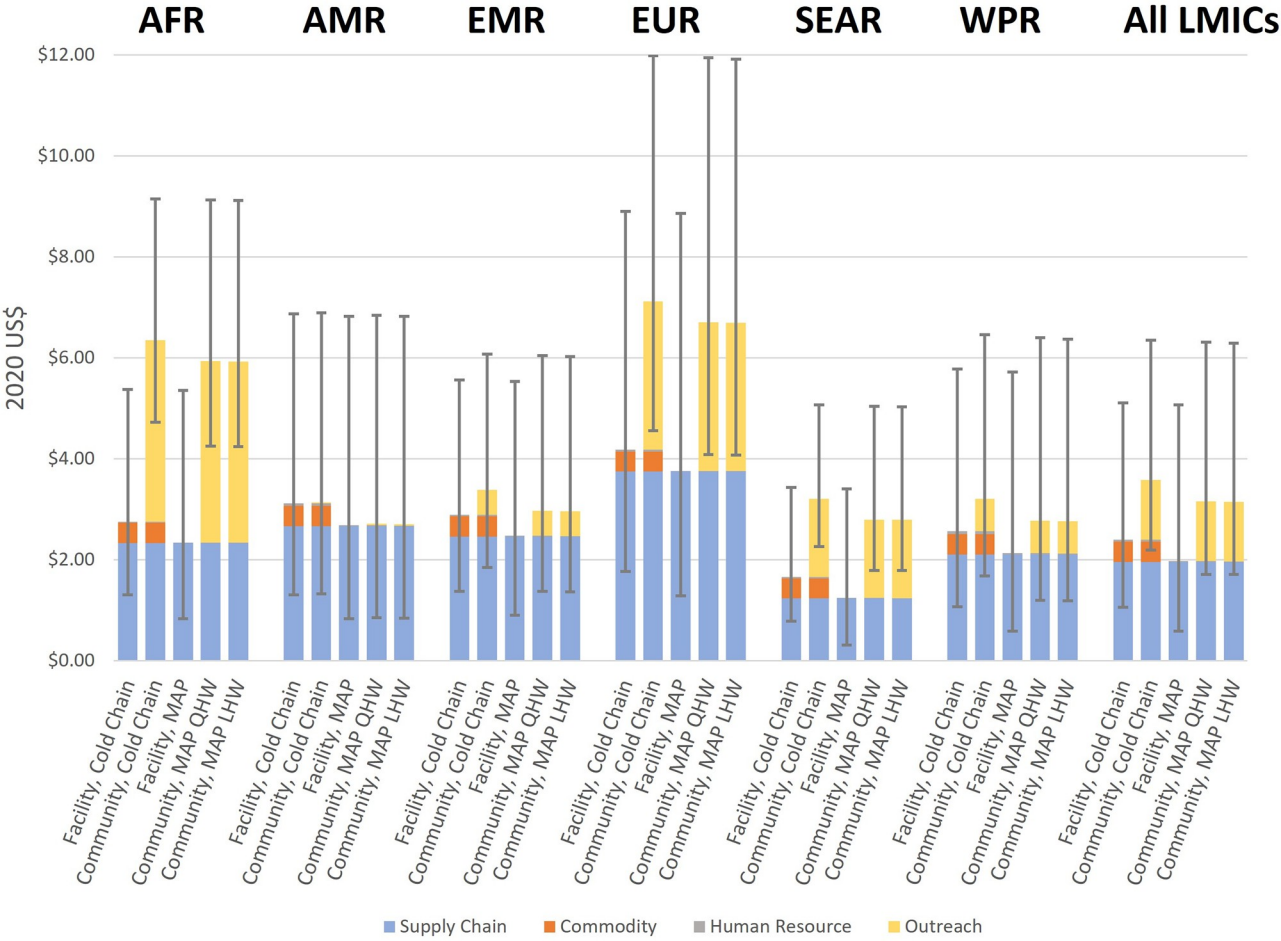
Component breakdown of modelled per-dose vaccination cost estimates for each WHO region. Note: Error bars represent uncertainty range used in analysis. MAP costs exclude commodity components. Abbreviations: AFR, African WHO Region AMR, American WHO Region; EMR, Eastern Mediterranean WHO Region; EUR, European WHO Region; LHW, lay health worker; LMICs, low- and middle-income countries; MAP, microarray patch; QHW, qualified health worker; SEAR, Southeast Asian WHO Region; US$, United States Dollar; WHO, World Health Organization; WPR, Western Pacific WHO Region.

For each vaccine modality, component and total costs in each WHO region can be seen in Fig 1, and further details provided in the supplement (Appendix 3 in S1 Text). Briefly, each vaccine modality was made up of four component costs. First, a supply chain component aimed to capture costs of vaccine transportation and storage to and within a health facility. Estimates were taken from a review by Portnoy and colleagues, with costs assumed equal for all modelled vaccination modalities [56]. Second, a commodity component aimed to capture costs of baseline vaccination coverage using vaccine vials (MDV and SDV), assuming a 3 MDV: 1 SDV procurement ratio at baseline. Price data were obtained from UNICEF-Supply Division (SD) data and included vaccines (MDV, US$0.25/dose; SDV, $0.55/dose), 0.5 mL autodisable syringes, disposal boxes, and wastage [9, 47, 57]. Although CPADs were excluded for this evaluation, their current UNICEF-SD price per dose ($1.65 for Uniject CPAD) was used to benchmark MAP price points for the analysis: $1.65 (equal to the price of a hepatitis B vaccine CPAD [58]), $3.30 (doubled CPAD price) and $5.00 as an upper limit. While true MAP price points are unclear due to no available commercial product, the upper threshold of US$5.00 represents a price approximately 10-fold that of an SDV, and procurement at higher costs in LMICs was deemed unfeasible. Third, a human resource component aimed to capture the worker time associated with vaccine administration: calculation of time needed to administer a vaccine was taken from a PATH time-and-motion study and then valued using methods described by Serje and colleagues [59, 60]. Fourth, an outreach component aimed to capture the additional costs of time and travel associated with vaccinating a birth in the community and were guided by costs from a modelling study by Nayagam and colleagues [61].

### Disease management costs and health utilities

Disease management costs included a one-off chronic hepatitis B diagnosis cost, plus recurrent (annual) costs of disease management. Costs were estimated for each LMIC, informed by the costing methodology used by Tordrup and colleagues [44]. For each stage of chronic disease, costs aimed to capture the regulatory testing, health resource utilization [54, 55], and antiviral therapy (tenofovir) where recommended [62].

DALYs were calculated as the sum of years life lost (YLL) and years lost to disability (YLD). YLDs were calculated as person time spent in each disease compartment, multiplied by the corresponding disability weight (Table 1) [42]. YLL were calculated relative to life expectancy at birth within each modelled setting and assumed as zero once surpassed [50].

### Model scenarios

An initial model baseline was run, using estimates of current HepB-BD coverage in each setting based on a vial presentation stored in the cold chain. Two potential MAP implementation scenarios are as follows:

1. **Additional coverage from MAPs**: MAPs facilitate a 1%, 5%, or 10% improvement in timely birth dose coverage, with vaccines administered to births previously not receiving any birth dose; additional coverage is delivered only by lay health workers to infants born in the community.
2. **Additional and replacement coverage from MAPs**: In addition to the above, MAPs also replace a proportion of existing birth dose coverage. Qualified health workers are assumed to use MAPs to replace some needle-and-syringe birth dose coverage due to the improved acceptability and increased ease of use of the MAP device. Replacement coverage could occur for births in both facility and community settings, with cost-effectiveness evaluated for MAPs that are used to replace 1%, 5%, and 10% of existing non-timely cold chain coverage. Use of MAPs to replace cold chain coverage was assumed to improve timeliness, modelled as a left shift to the next most prompt vaccine timing strata (i.e., from days 8–41 to days 3–7, from days 3–7 to day 2, and from day 2 to day 1).

### Model outcomes

Primary model outcomes were total lifetime costs (including vaccination costs and disease management costs) and total lifetime hepatitis B-related DALYs. For the three potential MAP price points evaluated ($1.65, $3.30, $5.00), incremental cost-effectiveness ratios (ICERs), or US$ per DALY averted, were calculated for implementation scenarios respective to the status quo baseline. For 51 (64%) LMICs with an available estimate, we evaluated cost-effectiveness against econometrically derived country-specific thresholds of willingness to pay per DALY averted [63]. Values reflected the opportunity costs of existing health expenditure within LMICs and were estimated using country-specific disease epidemiology, mortality, and demography. For all analyzed LMICs, cost-effectiveness was measured against proportional (0.5, 1.0, and 3.0 times) per capita GDP thresholds.

Uncertainty analysis was conducted using 1,000 Monte Carlo simulations for each model application, with outcome uncertainty presented as interquartile range (IQR). Where data was available with 95% confidence intervals (disease prevalence, costs, vaccine effectiveness, disability weights; Tables 1 and 2); uncertainty was drawn from triangular distributions to replicate the normal distribution. Uniform distributions were used for data available with a range (disease mortality, disease progression), or by applying ±5% error bounds for point estimates (vaccine coverage, all-cause mortality, facility births).

### Sensitivity analysis

Determinants of cost-effectiveness and impacts of MAP product profile assumptions were evaluated in a one-way sensitivity analysis, with MAP procurement price fixed at $1.65 per dose. Under a scenario where MAP price is fixed, despite being theoretical; the impact of other HepB-BD MAP unknowns such as the product size, cold chain requirements, and patch application times can be evaluated.

Within the vaccine supply chain, a worst-case scenario (MAPs are a higher unit size and require cold chain storage) and best-case scenario (MAPs completely bypass the cold chain) were approximated by respective doubling and halving of supply chain costs. Use of MAPs under a CTC approach could eliminate the need for peripheral cold chain and allow storage of vaccines closer to births in the community. As modelled outreach costs included a cold chain component, they were assumed the upper-bound estimate. We approximated savings from a CTC approach for MAPs as a 20% discount to the outreach cost component of community births attended by a qualified health worker (removal of preparation and carriage of a mobile cold chain device) and 50% discount for those attended by lay health workers (vaccines stored within the community, reducing transportation and travel time costs to and from health facilities). Some evidence suggests MAPs may need to adhere to the skin for as long as 120 seconds to administer a full vaccine dose; this is used as an upper-bound estimate for human resource costs within the sensitivity analysis.

For brevity, we present results from the aggregate (all LMICs) within the article; however, analysis was completed for all modelled settings (80 LMICs and six WHO regions; S1 File).

### Ethics approval

As this was a modelling study which used openly available data, ethics approval was not required.

## Results

Across the 80 LMICs currently providing universal hepatitis B birth dose vaccination, we estimate over the 2020 birth cohort lifetime mother-to-child hepatitis B transmission causes the loss of 1.5 (IQR: 1.2, 1.9) million DALYs (Table 3). Across this cohort, we estimate $164 (IQR: 137, 203) million was spent on vaccines delivered from vials stored in the cold chain and delivered by qualified health workers, while a discounted $400 (IQR: 287, 542) million would be spent on mother-to-child transmission attributable hepatitis B disease management over the lifetime.

**Table 3:** Regional model outputs.

	MAPs Administered	DALYs Averted (for MAPs additional DALYs averted vs baseline)	Disease Management Costs (in thousand US$)	Vaccination Costs (excluding MAP commodity costs; in thousand US$)	ICER, US$ per DALY averted (MAP procurement price = US$1.65 per dose)	ICER, US$ per DALY averted (MAP procurement price = US$3.30 per dose)	ICER, US$ per DALY averted (MAP procurement price = US$5.00 per dose)
**All LMICs (80 countries, 80.1 million births)**							
Baseline (MDV+SDV)	--	1,538,536 (1,224,957; 1,868,764)	399,541 (287,497; 541,512)	163,807 (136,555; 203,261)	--	--	--
+1% additional coverage with MAPs	49,162 (43,425; 55,272)	2,468 (1,936: 3,116)	398,795 (287,021; 540,606)	163,955 (136,714; 203,425)	-154.44 (-242.33; -86.88)	-119.64 (-204.17; -52.48)	-88.65 (-171.22; -15.44)
+1% additional and 1% replacement coverage with MAPs	194,369 (184,273; 204,183)	3,476 (2,785: 4,398)	398,507 (286,803; 540,317)	163,966 (136,808; 203,421)	-132.86 (-222.07; -59.75)	-41.04 (-126.66; 40.51)	54.40 (-34.88; 152.73)
+1% additional and 5% replacement coverage with MAPs	773,426 (746,364; 799,418)	7,552 (5,952: 9,643)	397,357 (286,139; 539,162)	163,647 (136,855; 202,958)	-101.92 (-205.27; -9.78)	64.17 (-41.07; 179.19)	233.64 (115.71; 386.49)
+1% additional and 10% replacement coverage with MAPs	1,498,144 (1,446,550; 1,546,363)	12,640 (9,818: 16,240)	395,919 (285,373; 537,718)	163,427 (136,590; 202,456)	-92.77 (-205.03; 11.27)	98.03 (-16.26; 233.87)	296.78 (161.09; 478.89)
**AFR (8 countries, 3.8 million births)**							
Baseline (MDV+SDV)	--	56,064 (42,854; 72,258)	15,481 (10,792; 22,087)	10,476 (9,155; 12,243)	--	--	--
+1% additional coverage with MAPs	2,225 (1,986; 2,496)	101 (75: 133)	15,453 (10,773; 22,053)	10,494 (9,171; 12,257)	-92.16 (-201.47; -13.73)	-55.50 (-166.86; 30.24)	-21.66 (-130.07; 69.32)
+1% additional and 1% replacement coverage with MAPs	10,113 (9,640; 10,613)	153 (114: 199)	15,438 (10,767; 22,030)	10,487 (9,175; 12,249)	-80.72 (-191.15; 0.08)	22.29 (-87.26; 130.40)	133.02 (20.54; 272.46)
+1% additional and 5% replacement coverage with MAPs	41,561 (40,107; 43,061)	353 (257: 459)	15,378 (10,736; 21,937)	10,479 (9,162; 12,227)	-76.88 (-197.17; 42.21)	122.57 (-15.35; 288.8)	321.83 (155.66; 560.81)
+1% additional and 10% replacement coverage with MAPs	80,966 (78,115; 83,722)	604 (431: 782)	15,330 (10,687; 21,821)	10,483 (9,155; 12,201)	-70.32 (-200.06; 59.17)	154.30 (0.56; 341.24)	382.78 (191.32; 660.72)
**AMR (20 countries, 9.5 million births)**							
Baseline (MDV+SDV)	--	55,751 (44,875; 70,713)	16,497 (11,809; 22,240)	22,192 (17,804; 27,614)	--	--	--
+1% additional coverage with MAPs	3,147 (2,184; 4,035)	40 (27: 57)	16,487 (11,798; 22,220)	22,196 (17,819; 27,629)	93.85 (-25.57; 194.95)	222.93 (91.97; 344.21)	346.32 (205.57; 502.34)
+1% additional and 1% replacement coverage with MAPs	16,271 (14,997; 17,658)	65 (49: 87)	16,481 (11,791; 22,210)	22,202 (17,808; 27,609)	196.69 (-34.35; 447.40)	608.14 (351.50; 933.75)	1055.11 (722.42; 1475.02)
+1% additional and 5% replacement coverage with MAPs	68,722 (66,041; 71,660)	165 (128: 220)	16,456 (11,771; 22,161)	22,204 (17,793; 27,586)	314.96 (-101.21; 767.71)	993.27 (530.40; 1583.73)	1706.78 (1169.13; 2466.75)
+1% additional and 10% replacement coverage with MAPs	134,390 (129,618; 139,527)	290 (221: 385)	16,424 (11,749; 22,105)	22,169 (17,809; 27,482)	346.49 (-132.48; 847.81)	1095.57 (576.46; 1787.89)	1898.83 (1286.29; 2783.89)
**EMR (9 countries, 8.0 million births)**							
Baseline (MDV+SDV)	--	60,047 (49,532; 73,416)	16,658 (11,939; 22,503)	19,030 (16,129; 22,450)	--	--	--
+1% additional coverage with MAPs	5,461 (4,814; 6,057)	110 (88: 139)	16,626 (11,916; 22,458)	19,047 (16,149; 22,469)	-24.53 (-121.47; 64.16)	55.96 (-37.94; 150.66)	140.42 (41.43; 246.41)
+1% additional and 1% replacement coverage with MAPs	20,532 (19,501; 21,624)	155 (124: 193)	16,610 (11,909; 22,438)	19,052 (16,144; 22,457)	26.43 (-81.50; 156.53)	247.93 (136.74; 408.11)	478.51 (333.37; 674.99)
+1% additional and 5% replacement coverage with MAPs	80,819 (77,872; 83,878)	328 (258: 419)	16,563 (11,877; 22,359)	19,044 (16,107; 22,421)	113.17 (-62.67; 325.57)	515.96 (320.31; 813.31)	947.82 (686.60; 1345.57)
+1% additional and 10% replacement coverage with MAPs	156,143 (150,592; 161,987)	542 (417: 702)	16,511 (11,832; 22,277)	19,067 (16,130; 22,391)	139.50 (-65.10; 396.23)	616.13 (376.28; 977.36)	1122.17 (810.35; 1594.06)
**EUR (17 countries, 4.3 million births)**							
Baseline (MDV+SDV)	--	39,047 (30,369; 48,171)	10,976 (7,741; 15,549)	20,043 (16,270; 24,527)	--	--	--
+1% additional coverage with MAPs	176 (103; 262)	10 (6: 15)	10,971 (7,738; 15,543)	20,044 (16,272; 24,528)	-117.37 (-222.77; -39.29)	-86.94 (-190.94; -9.02)	-55.03 (-158.45; 26.80)
+1% additional and 1% replacement coverage with MAPs	9,112 (8,689; 9,476)	82 (62: 106)	10,955 (7,724; 15,518)	20,041 (16,283; 24,528)	-139.88 (-296.74; 31.38)	50.19 (-109.37; 234.74)	244.49 (68.00; 464.31)
+1% additional and 5% replacement coverage with MAPs	44,682 (42,947; 46,292)	362 (282: 477)	10,871 7,668; 15,415)	20,051 (16,355; 24,458)	-137.89 (-307.21; 43.56)	63.41 (-102.56; 262.89)	277.65 (85.67; 527.47)
+1% additional and 10% replacement coverage with MAPs	89,172 (85,774; 92,350)	716 (555: 943)	10,762 (7,592; 15,273)	20,090 (16,340; 24,362)	-137.67 (-308.18; 47.74)	65.94 (-102.28; 266.47)	283.02 (88.47; 533.51)
**SEAR (8 countries, 32.0 million births)**							
Baseline (MDV+SDV)	--	593,396 (482,632; 710,357)	151,496 (111,808; 164,622)	38,584 (32,995; 46,052)	--	--	--
+1% additional coverage with MAPs	44,990 (41,208; 48,437)	1,637 (1,313: 2,026)	151,135 (111,511; 164,544)	38,756 (33,144; 46,224)	-118.41 (-214.19; -52.10)	-73.21 (-164.25; -3.40)	-26.26 (-115.66; 44.98)
+1% additional and 1% replacement coverage with MAPs	94,104 (89,158; 98,851)	1,870 (1,529: 2,305)	151,088 (111,459; 164,243)	38,756 (33,128; 46,191)	-99.13 (-194.04; -32.52)	-13.67 (-106.97; 54.72)	69.39 (-21.79; 148.59)
+1% additional and 5% replacement coverage with MAPs	290,264 (279,364; 300,658)	2,859 (2,322: 3,587)	150,899 (111,252; 163,298)	38,703 (33,076; 46,092)	-55.98 (-158.18; 17.41)	108.76 (5.68; 210.32)	281.17 (161.13; 410.26)
+1% additional and 10% replacement coverage with MAPs	534,737 (516,682; 553,714)	4,103 (3,230: 5,207)	150,663 (110,993; 162,011)	38,611 (33,033; 45,936)	-36.38 (-140.73; 49.63)	181.71 (57.77; 308.33)	412.14 (257.81; 578.75)
**WPR (18 countries, 22.6 million births)**							
Baseline (MDV+SDV)	--	464,860 (368,293; 569,978)	119,310 (87,269; 164,622)	61,059 (48,822; 76,622)	--	--	--
+1% additional coverage with MAPs	2,288 (1,760; 3,022)	229 (166: 312)	119,260 (87,226; 164,544)	61,062 (48,829; 76,635)	-210.88 (-312.10; -138.05)	-192.44 (-293.77; -121.80)	-174.36 (-274.86; -104.83)
+1% additional and 1% replacement coverage with MAPs	48,072 (46,177; 50,051)	889 (699: 1,119)	119,101 (87,115; 164,243)	61,041 (48,849; 76,579)	-179.82 (-280.96; -99.09)	-89.17 (-185.74; -0.15)	1.48 (-92.79; 96.61)
+1% additional and 5% replacement coverage with MAPs	230,093 (222,998; 238,518)	3,455 (2,734: 4,444)	118,464 (86,582; 163,298)	60,950 (48,914; 76,433)	-174.85 (-277.08; -82.50)	-67.69 (-162.62; 39.86)	49.94 (-56.02; 167.89)
+1% additional and 10% replacement coverage with MAPs	457,631 (443,864; 474,500)	6,660 (5,232: 8,549)	117,542 (85,850; 162,011)	60,845 (49,108; 76,315)	-173.83 (-278.00; -78.72)	-63.58 (-158.48; 45.77)	59.05 (-49.61; 180.21)

Negative ICERs indicate cost-savings over the cohort lifetime. Parenthesised uncertainty the Interquartile Range (IQR) of 1000 model simulations. All costs presented in 2020 US$.

*Abbreviations*: AFR: African WHO Region, AMR: American WHO Region, DALY: Disability Adjusted Life Year, EMR: Eastern Mediterranean WHO Region, EUR: European WHO Region, ICER: Incremental Cost-Effectiveness Ratio (US$ per DALY averted), LMIC: Low-and middle-income country, MAP: Microarray Patch, MDV: Multiple Dose Vial, SDV: Single Dose Vial, SEAR: Southeast Asian WHO Region, US$: United States Dollars, WHO: World Health Organization, WPR: Western Pacific WHO Region

Data for individual LMICs available in the S1 File.

**Fig 2:**
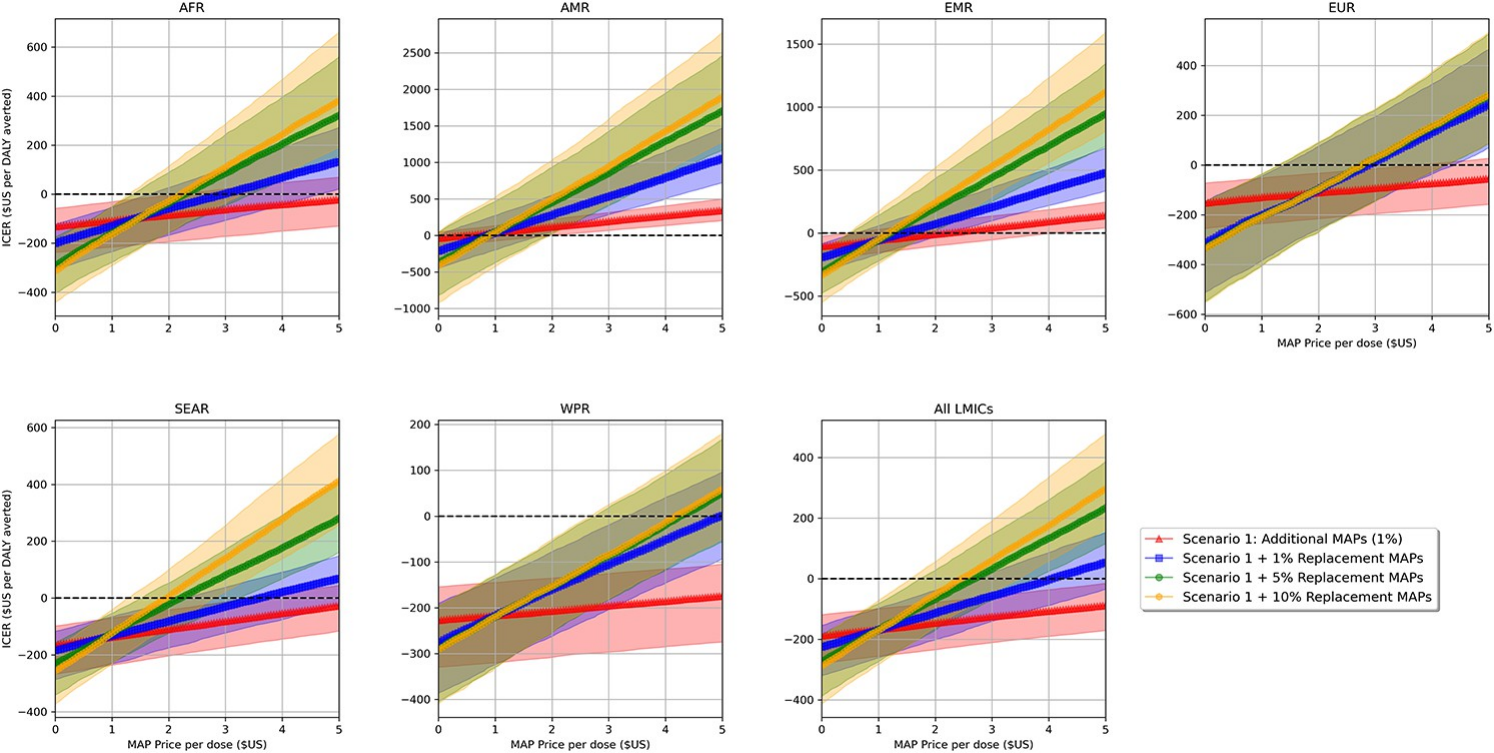
Incremental cost-effectiveness of MAPs at increasing price-per-unit values in each WHO region. Note: Shading represents interquartile range of 1000 model simulations. As ICER increases (ascends along the Y-axis), cost-effectiveness of MAPs decreases. Above dotted line indicates additional costs per DALY averted over the cohort lifetime, below indicates costs-saved per DALY averted over the cohort lifetime. Abbreviations: AFR: African WHO Region, AMRO: American WHO Region, DALY: disability-adjusted life years, EMRO: Eastern Mediterranean WHO region, EURO: European WHO Region, ICER: incremental cost-effectiveness ratio, LMIC: low- and middle-income countries, MAP: microarray patch, SEARO: Southeast Asian WHO Region, WHO: World Health Organization, WPRO: Western Pacific WHO Region.

We estimate that MAPs could be used to administer between 49 (IQR: 43, 55) thousand and 1.9 (IQR: 1.8, 2.0) million hepatitis B birth dose vaccinations across the 80 modelled LMICs, averting between 2.5 (IQR: 1.9, 3.1) thousand and 38 (IQR: 28, 44) thousand DALYs over the cohort lifetime (Table 2 and Table G in S1 Text). Expanding coverage to previously unvaccinated births in the community (scenario 1) was the most efficient use of MAPs, with incremental cost-effectiveness unaltered by the level of coverage provided (i.e., ICERs remained constant; Table G in S1 Text). In comparison, the efficiency of MAPs generally decreased as more were used to replace existing hepatitis B birth dose coverage (Fig 2; Table 3). However, a trade-off was observed between increasing new coverage and increasing replacement coverage; whereby efficiency of replacement coverage was greater when paired with higher incremental coverage expansions (Fig C and Table G in S1 Text). Thus, we conservatively present results where MAPs only provide a 1% gain in additional coverage. If MAPs were used to provide more than 1% additional coverage, model scenario 2 would be even more cost-effective or cost-saving than presented (S1 File).

While at a price of $1.65 per unit MAPs are more expensive per dose than comparator vial vaccines, when used to expand hepatitis B birth dose coverage in the community (scenario 1), averted disease and associated management costs generated overall cost-savings in 5 (83%) WHO regions and 50 (63%) LMICs. At a procurement price of $3.30 per MAP, cost savings were seen in 4 (67%) of the WHO regions and 41 (51%) of the LMICs, further reducing to 4 (67%) and 36 (45%), respectively, at $5.00 per MAP. Even though cost-savings reduced as the price of MAPs increased, analysis against estimates of national willingness-to-pay thresholds ($ per DALY averted) suggested that, for prices up to $5.00 per MAP, use under model scenario 1 could be considered cost-effective in all but one modelled LMIC (Fig B in S1 Text).

Although less efficient, cost-savings were still observed in multiple analysed settings when MAPs were also used to replace a proportion of existing hepatitis B birth dose coverage by qualified health workers (scenario 2). At a procurement price of $1.65 per MAP, replacing 1% of existing coverage in addition to expanding coverage in communities by 1%, generated cost-savings in at least 4 (67%) WHO regions and 53 (66%) modelled LMICs. Comparatively, when replacing 10% of existing coverage at a procurement price of US$5.00 per MAP, cost-savings were seen in no WHO regions and only 12(15%) LMICs. Evaluation against national willingness-to-pay thresholds indicated that at $1.65 per MAP, model scenario 2 could be considered cost-effective in all but a single LMIC; while at a price of US$3.30 or US$5.00 per MAP, use may no longer be considered cost effective in at least seven and up to fourteen LMICs (Fig B in S1 Text).

**Fig 3:**
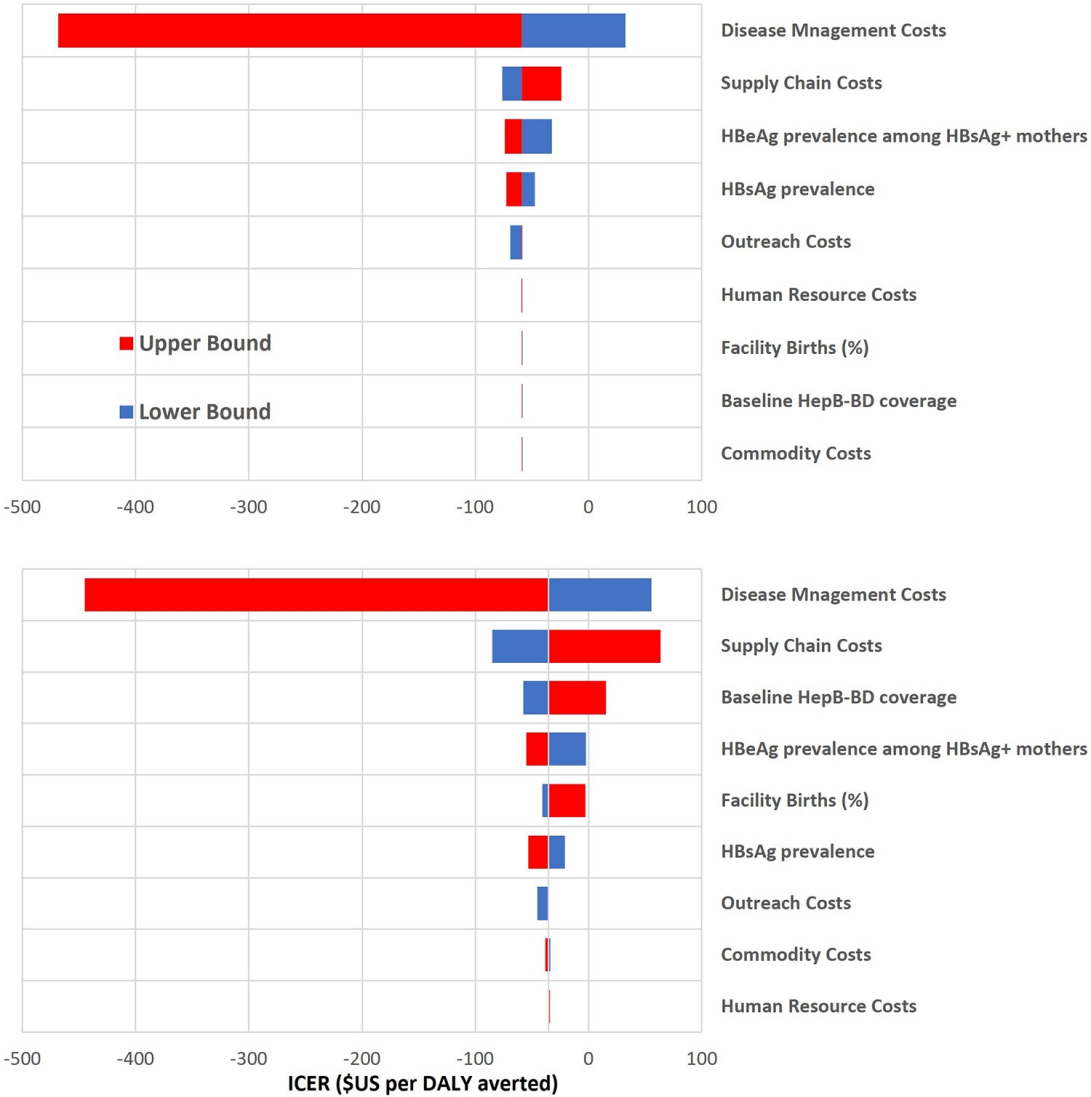
Tornado plots displaying outcomes of one-way sensitivity analysis holding MAP price constant. Notes: Top: 1% additional coverage (scenario 1) within all LMICs. Bottom: Scenario 1 + 1% replacement coverage with MAPs in LMICs (scenario 2). HBsAg and HBeAg upper and lower bounds correspond with values given in Table 2. Facility birth and baseline HepB-BD coverage’s lower bound = 5%, upper bound = 99%. MAPs could reduce outreach costs by 20% for community births vaccinated by a qualified health worker and 50% for community births vaccinated by a trained lay health worker; upper-bound vaccine administration time is estimated at two minutes (120 seconds). Commodity costs used upper and lower bounds of estimates (see Table C in S1 Text), while supply chain costs were doubled or halved for MAPs, as cost impact remains uncertain. Abbreviations: DALY: disability-adjusted life years, HBeAg: hepatitis B envelope antigen, HBsAg: hepatitis B surface antigen, HepB-BD: hepatitis B birth dose, ICER: incremental cost-effectiveness ratio, LMICs: low-and middle-income countries, MAP: microarray patch.

Despite some heterogeneity across individual LMICs in the results of the one-way sensitivity analyses (S1 File), for the majority it was disease management costs that were the main driver of cost-effectiveness when holding MAP price constant (Fig 3). Assumptions regarding MAP administration time (human resource costs) and benefits of ambient storage (outreach costs) only showed modest impacts on cost-effectiveness. Other drivers related to the vaccine, including supply chain costs had more effect on the model outputs when MAPs were assumed to replace existing coverage (scenario 2). In contrast, baseline coverage levels did not alter cost-effectiveness when MAPs were only assumed to expand coverage and not replace existing coverage (scenario 1). Further, when a baseline scenario with traditional vial vaccines being stored under the CTC was investigated, findings showed no meaningful difference to cost-effectiveness outcomes when using MAPs to expand hepatitis B birth dose coverage (Fig G and Table I in S1 Text).

## Discussion

Using a mathematical model, we demonstrate that MAPs—even at much higher procurement prices relative to current vial presentations—could provide a cost-saving or highly cost-effective mechanism for increasing the reach of hepatitis B birth dose vaccination. Our findings indicate that use of MAPs to increase birth dose coverage to previously unreached births would be their most cost-effective use, saving costs attributable to mother-to-child hepatitis B transmission in 50, 41 and 36 analysed LMICs at respective commodity costs of US$1.67, US$3.30, and US$5.00 per dose. Although less efficient, use of MAPs to also replace existing hepatitis B birth dose vaccination was found to remain cost-effective in most analysed LMICs. Our findings are consistent with previous economic evaluations demonstrating value for money provided by hepatitis B birth dose vaccination [64], with the WHO recently describing it as the most efficient intervention for combatting the ongoing hepatitis B burden [65].

This work adds to previous evaluations which show both the CTC approach, and use of CPADs, as highly cost-effective mechanisms to enhance birth dose vaccination coverage in LMICs [14, 15]. We demonstrate that MAPs would likely work synergistically with any future CTC licenced vaccines and recognise development of these remain important. However, historic uptake of other CTC licensed vaccines has been slow—due to numerous reasons such as cost and fear of health worker confusion [10, 18]–and in isolation would not address the problem of qualified health workers needing to reach births outside of health facilities. MAPs could both bypass standard cold chain storage constraints and allow lay health workers within the community to provide timely birth dose vaccinations to previously unreachable births. CPADs have successfully been piloted to overcome this barrier [66]; yet uptake remains slow in most settings due to cost, cold chain volumes, market availability and lack of support from procurers or funding bodies. MAPs are likely to surpass CPADs on several product fronts (size, no sharps waste, ease of use), and due to ongoing catalytic work being undertaken by global stakeholders. Specifically, MAPs were prioritized by the Vaccine Innovation Prioritisation Strategy Alliance, which has developed action plans to accelerate their development for several vaccine applications, is incentivizing investment in MAPs, and is engaging in activities that will position MAPs for greater uptake once available [24, 25, 31, 67].

Our analysis showed that use of MAPs to expand birth dose coverage in the community provided greater value than when used concurrently to replace a proportion of existing coverage. Consistent with previous hepatitis B birth dose modelling, our findings support the use of more expensive vaccine modalities, such as MAPs, to reach births where current presentations may not be able to reach [14]. Nevertheless, implementation experiences with CPADs indicate health workers may prefer use of combination products such as MAPs, due to the speed and simplicity they afford [20, 66]. While we show this decreases efficiency of MAPs for the hepatitis B birth dose; this practice, when paired alongside vaccination of previously unreached births, remained cost-effective in most analysed LMICs. We also note that reductions in efficiency were dampened as more previously unvaccinated births received a timely birth dose, potentially supporting use in this manner if additional coverage is seen as the priority.

It remains unclear how individual LMICs would benchmark cost-effectiveness, what with many other factors influencing decisions to adopt new health interventions. In this analysis we have assessed different use cases and price points for MAPs, reporting whether they are likely to be either cost saving or cost-effective as benchmarked against estimated national willingness-to-pay thresholds per unit of health. However, consideration also needs to be given to affordability: MAPs cost savings would be accrued over the lifetime of a birth cohort, while MAPs procurement presents an additional upfront cost of size, dependent on the population in need. Hence, factors such as disease control priorities (e.g., relative importance of hepatitis B or infectious diseases within the health budget allocation) will serve as major considerations in the affordability of MAPs within LMICs, once available. Nevertheless, the estimates of the ICERs of MAPs for different use cases and price points provided by this study enable them to be objectively compared to other key interventions.

First, hepatitis B birth dose MAPs are still a developing technology, and currently the product profile and programmatic impact are yet to be determined, key inputs that would impact the cost-effectiveness. Given these uncertainties in model inputs, our analysis conducted scenario and sensitivity analyses to identify the key drivers influencing this early-stage value proposition but revisions to this model may be warranted as the product progresses through development stages and during programmatic introduction. Second, our model did not account for the impact that a birth dose vaccination may have on early life horizontal transmission, potentially underestimating the impact and cost-effectiveness of expanding coverage. However, in settings where horizontal transmission is believed to be the key driver of ongoing burden, rates of mother-to-child transmission were lower within the model [51]. Third, we did not include the cost impact of other mother-to-child-transmission strategies, such as antivirals in late pregnancy or use of passive immunoprophylaxis (hepatitis B immune globulin); but within many LMICs, coverage with these interventions is low or unfeasible [7, 68]. Lastly, our results are only representative of 80 LMICs with existing HepB-BD vaccination coverage and do not provide any evidence to support MAPs for introducing a birth dose vaccination program, although it would likely be cost saving or cost-effective. In particular, the regional analysis excludes data from countries without birth dose programs. Therefore, the weighted average parameters are representative not of each region but of a subset of countries within each region only (each region can be thought of as an aggregate of countries where data are available).

## Conclusion

These findings suggest that use of MAPs to expand hepatitis B birth dose vaccination coverage in LMICs is likely to be cost saving or cost-effective. While it is likely to be several years before a relevant MAP product is approved for use and on the market, this work supports ongoing research and development of MAPs to prevent mother-to-child transmission of hepatitis B.

## Supporting information

S1 Text In the document supplementary material, additional details on several aspects of the study are provided including model structure, costing methodology, results, and sensitivity analysis. (PDF)

S1 File Supplemental excel workbook containing input data, outputs, and sensitivity analysis for all 80 modelled LMICs and six WHO global regions. (XLSX)
