# Supplementary material for: Evaluating the potential cost-effectiveness of microarray patches to expand access to hepatitis B birth dose vaccination in low-and middle-income countries: A modelling study
Source: PLOS Glob Public Health. 2022 May 23;2(5):e0000394. doi: 10.1371/journal.pgph.0000394 (PMC10021446; doi:10.1371/journal.pgph.0000394)
Supplement: S1 Text — (PDF) [file pgph.0000394.s001.pdf]

Evaluating the potential cost-effectiveness of microarray patches for expanding hepatitis B birth dose vaccination in low-and middle-income countries: A modelling study

Supplemental Materials

Christopher P Seaman, Mercy Mvundura, Collrane Frivold, Chris Morgan, Courtney Jarrahian, Jess Howell, Margaret Hellard, Nick Scott

Appendix 1: Regional aggregation of low and middle-income countries within the model

Analysis focused on low-and middle-income countries (LMICs) providing hepatitis B birth dose vaccination for all newborns in the WHO/UNICEF estimates of National Immunization Coverage (WUENIC) [1]. Coverage estimates for LMICs could be from either 2019 or 2020, with the most recent in each LMIC used for modelling. From these countries, we modelled the six WHO regions, plus a composite “All LMICs” region encompassing all 77 LMICs. Population-weighted parameter averages (using 2020 births) [2] from relevant country subsets were used for regional analysis, excluding those with missing data.

Each modelled LMIC also needed to be aggregated into a Global Burden of Disease (GBD) world region for estimation of HBeAg prevalence among 15-49y females (reproductive age) [3], GBD Super Regions for estimates of costs associated with vaccination outreach [4], and World Bank income classification for estimation of human resource costs [5]. Missing data for modelled LMICs was imputed using the relevant WHO regional average (Table 2, Main Text).

Table A: Regional classification and basic epidemiological profile of modelled LMICs

**LMIC**	**WHO Region** [6]	**GBD World Region** [7]	**GBD Super Region** [7]	**World Bank Income Classification#** [8]	**Hepatitis B birth dose coverage (WUENIC) [1]**
Afghanistan	EMR	North Africa and Middle East	North Africa and Middle East	Low Income	38%
Albania	EUR	Central Europe	Central Europe, Eastern Europe and Central Asia	Upper Middle Income	99%
Algeria	AFR	North Africa and Middle East	North Africa and Middle East	Upper Middle Income	99%
Argentina	AMR	Southern Latin America	Latin America and Caribbean	Upper Middle Income	72%
Armenia	EUR	Central Asia	Central Europe, Eastern Europe and Central Asia	Upper Middle Income	97%

Azerbaijan	EUR	Central Asia	Central Europe, Eastern Europe and Central Asia	Upper Middle Income	98%
Belarus	EUR	Eastern Europe	Central Europe, Eastern Europe and Central Asia	Upper Middle Income	97%
Belize	AMR	Caribbean	Latin America and Caribbean	Upper Middle Income	67%
Benin	AFR	Western Sub-Saharan Africa	Sub-Saharan Africa	Low Income	21%
Bhutan	SEAR	South Asia	South Asia	Lower Middle Income	85%
Brazil	AMR	Tropical Latin America	Latin America and Caribbean	Upper Middle Income	63%
Bulgaria	EUR	Central Europe	Central Europe, Eastern Europe and Central Asia	Upper Middle Income	98%
Cabo Verde	AFR	Western Sub-Saharan Africa	Sub-Saharan Africa	Lower Middle Income	96%
Cambodia	WPR	Southeast Asia	Southeast Asia, East Asia and Oceania	Lower Middle Income	94%
China	WPR	East Asia	Southeast Asia, East Asia and Oceania	Upper Middle Income	95%
Colombia	AMR	Central Latin America	Latin America and Caribbean	Upper Middle Income	88%
Costa Rica	AMR	Central Latin America	Latin America and Caribbean	Upper Middle Income	87%
Côte d'Ivoire	AFR	Western Sub-Saharan Africa	Sub-Saharan Africa	Lower Middle Income	62%
Cuba	AMR	Caribbean	Latin America and Caribbean	Upper Middle Income	99%
Djibouti	EMR	Eastern Sub-Saharan Africa	Sub-Saharan Africa	Lower Middle Income	77%
Dominica	AMR	Caribbean	Latin America and Caribbean	Upper Middle Income	99%
Dominican Republic (the)	AMR	Caribbean	Latin America and Caribbean	Upper Middle Income	66%
Ecuador	AMR	Andean Latin America	Latin America and Caribbean	Upper Middle Income	62%
Egypt	EMR	North Africa and Middle East	North Africa and Middle East	Lower Middle Income	92%
El Salvador	AMR	Central Latin America	Latin America and Caribbean	Lower Middle Income	75%
Fiji	WPR	Oceania	Southeast Asia, East Asia and Oceania	Upper Middle Income	99%
Georgia	EUR	Central Asia	Central Europe, Eastern Europe and Central Asia	Upper Middle Income	97%
Grenada	AMR	Caribbean	Latin America and Caribbean	Upper Middle Income	92%
Guatemala	AMR	Central Latin America	Latin America and Caribbean	Upper Middle Income	48%
Guyana	AMR	Caribbean	Latin America and Caribbean	Upper Middle Income	70%
Honduras	AMR	Central Latin America	Latin America and Caribbean	Lower Middle Income	71%
India	SEAR	South Asia	South Asia	Lower Middle Income	54%
Indonesia	SEAR	Southeast Asia	Southeast Asia, East Asia and Oceania	Lower Middle Income	73%
Iran (Islamic Republic of)	EMR	North Africa and Middle East	North Africa and Middle East	Upper Middle Income	95%
Iraq	EMR	North Africa and Middle East	North Africa and Middle East	Upper Middle Income	47%

Kazakhstan	EUR	Central Asia	Central Europe, Eastern Europe and Central Asia	Upper Middle Income	93%
Kiribati	WPR	Oceania	Southeast Asia, East Asia and Oceania	Lower Middle Income	91%
Korea (the Democratic People's Republic of)	SEAR	East Asia	Southeast Asia, East Asia and Oceania	Low Income	99%
Kyrgyzstan	EUR	Central Asia	Central Europe, Eastern Europe and Central Asia	Lower Middle Income	95%
Lao People's Democratic Republic (the)	WPR	Southeast Asia	Southeast Asia, East Asia and Oceania	Lower Middle Income	58%
Lebanon	EMR	North Africa and Middle East	North Africa and Middle East	Upper Middle Income	77%
Malaysia	WPR	Southeast Asia	Southeast Asia, East Asia and Oceania	Upper Middle Income	99%
Maldives	SEAR	Southeast Asia	Southeast Asia, East Asia and Oceania	Upper Middle Income	99%
Marshall Islands (the)	WPR	Oceania	Southeast Asia, East Asia and Oceania	Upper Middle Income	98%
Mexico	AMR	Central Latin America	Latin America and Caribbean	Upper Middle Income	52%
Micronesia (Federated States of)	WPR	Oceania	Southeast Asia, East Asia and Oceania	Lower Middle Income	64%
Moldova (the Republic of)	EUR	Eastern Europe	Central Europe, Eastern Europe and Central Asia	Lower Middle Income	95%
Mongolia	WPR	Central Asia	Central Europe, Eastern Europe and Central Asia	Lower Middle Income	98%
Morocco	EMR	North Africa and Middle East	North Africa and Middle East	Lower Middle Income	41%
Myanmar	SEAR	Southeast Asia	Southeast Asia, East Asia and Oceania	Lower Middle Income	21%
Namibia	AFR	Southern Sub-Saharan Africa	Sub-Saharan Africa	Upper Middle Income	81%
Nauru	WPR	Oceania	Southeast Asia, East Asia and Oceania	Upper Middle Income	99%
North Macedonia	EUR	Central Europe	Central Europe, Eastern Europe and Central Asia	Upper Middle Income	97%
Palestine, State of	EMR	North Africa and Middle East	North Africa and Middle East	Lower Middle Income	99%
Papua New Guinea	WPR	Oceania	Southeast Asia, East Asia and Oceania	Lower Middle Income	24%
Peru	AMR	Andean Latin America	Latin America and Caribbean	Upper Middle Income	75%
Philippines (the)	WPR	Southeast Asia	Southeast Asia, East Asia and Oceania	Lower Middle Income	53%
Romania	EUR	Central Europe	Central Europe, Eastern Europe and Central Asia	Upper Middle Income	97%
Saint Lucia	AMR	Caribbean	Latin America and Caribbean	Upper Middle Income	86%
Saint Vincent and the Grenadines	AMR	Caribbean	Latin America and Caribbean	Upper Middle Income	96%
Samoa	WPR	Oceania	Southeast Asia, East Asia and Oceania	Upper Middle Income	79%

São Tomé and Principe	AFR	Western Sub-Saharan Africa	Sub-Saharan Africa	Lower Middle Income	95%
Senegal	AFR	Western Sub-Saharan Africa	Sub-Saharan Africa	Lower Middle Income	86%
Serbia	EUR	Central Europe	Central Europe, Eastern Europe and Central Asia	Upper Middle Income	99%
Solomon Islands	WPR	Oceania	Southeast Asia, East Asia and Oceania	Lower Middle Income	70%
Suriname	AMR	Caribbean	Latin America and Caribbean	Upper Middle Income	79%
Tajikistan	EUR	Central Asia	Central Europe, Eastern Europe and Central Asia	Low Income	99%
Thailand	SEAR	Southeast Asia	Southeast Asia, East Asia and Oceania	Upper Middle Income	99%
Timor-Leste	SEAR	Southeast Asia	Southeast Asia, East Asia and Oceania	Lower Middle Income	72%
Tonga	WPR	Oceania	Southeast Asia, East Asia and Oceania	Upper Middle Income	99%
Tunisia	EMR	North Africa and Middle East	North Africa and Middle East	Lower Middle Income	74%
Turkey	EUR	North Africa and Middle East	North Africa and Middle East	Upper Middle Income	99%
Turkmenistan	EUR	Central Asia	Central Europe, Eastern Europe and Central Asia	Upper Middle Income	99%
Tuvalu	WPR	Oceania	Southeast Asia, East Asia and Oceania	Upper Middle Income	99%
Ukraine	EUR	Eastern Europe	Central Europe, Eastern Europe and Central Asia	Lower Middle Income	69%
Uzbekistan	EUR	Central Asia	Central Europe, Eastern Europe and Central Asia	Lower Middle Income	97%
Vanuatu	WPR	Oceania	Southeast Asia, East Asia and Oceania	Lower Middle Income	59%
Venezuela (Bolivarian Republic of)	AMR	Central Latin America	Latin America and Caribbean	Upper Middle Income	50%
Viet Nam	WPR	Southeast Asia	Southeast Asia, East Asia and Oceania	Lower Middle Income	82%
Zambia	AFR	Eastern Sub-Saharan Africa	Sub-Saharan Africa	Lower Middle Income	94%

AFR: African WHO Region, AMR: American WHO Region, EMR: Eastern Mediterranean WHO Region, EUR: European WHO Region, SEAR: Southeast Asian WHO Region, WPR: Western Pacific WHO Region.

World Bank Income Classification is for the 2020 calendar year

Appendix 2: Additional disease and vaccination model detail

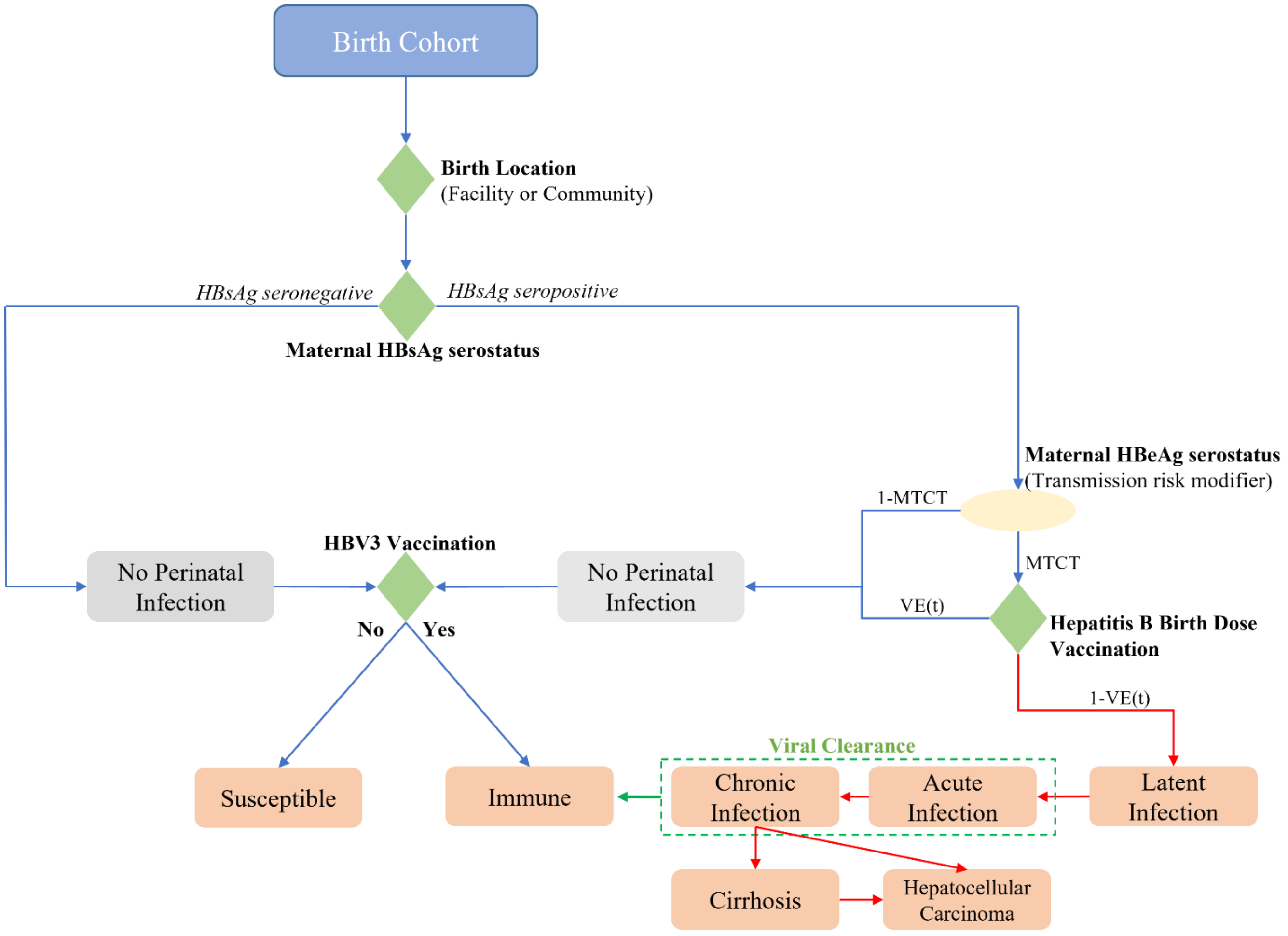

Fig A: Perinatal hepatitis B transmission and progression model. **Note:** Horizontal (non-MTCT) transmission is explicitly excluded from the model.

HBsAg = hepatitis B surface antigen, HBeAg = hepatitis B envelope antigen, MTCT=mother-to-child transmission risk, VE(t) = Vaccine effectiveness at each modelled postpartum time-strata (day 1, day 2, days 3-7, days 8-41 and unvaccinated).

Baseline distribution of hepatitis B birth dose coverage: facilities vs community

To account for reported coverage inequities amongst births occurring outside of health facilities (i.e., in the community) within the model [9, 10], baseline estimates of hepatitis B birth dose vaccination coverage were weighted according to the below formula. This was required as national estimates of hepatitis B birth dose coverage are reflective of coverage across an entire annual birth cohort, and hence do not capture any heterogeneity across birth locations.

A weighting factor (w.f.) of 2 was used within the model, consistent with observational data indicating odds of vaccination of births at home are approximately half that of those occurring within health facilities [11, 12].

Facility:

$$Facility\ Coverage(BD) = \begin{cases} BD + BD\left(\frac{wf-1}{wf+1}(2 \times Community)\right) & if\ BD \leq 50\% \\ BD + (1-BD)\left(\frac{wf-1}{wf+1}(2 \times Community)\right) & if\ BD > 50\% \end{cases}$$

Community:

$$Community\ Coverage(BD) = \begin{cases} BD - BD\left(\frac{wf-1}{wf+1}(2 \times Facility)\right) & if\ BD \leq 50\% \\ BD - (1-BD)\left(\frac{wf-1}{wf+1}(2 \times Facility)\right) & if\ BD > 50\% \end{cases}$$

BD: Baseline hepatitis B birth dose vaccine coverage, *wf*: Weighting Factor; odds of facilitated vaccination against community, *Facility/Community*: Proportion of facility/community births

Baseline distribution of hepatitis B birth dose coverage: timing of delivery

It was assumed that birth dose vaccination timing would be prompter for facility births, as compared to those in the community, given vaccine availability and vaccine access considerations. At baseline, we assumed that 80% vaccinations given in a health facility would be timely (<24 hours); a midpoint average between observed values of 64% and 90% [13, 14]. Remaining doses were

uniformly distributed among the non-timely vaccination strata (i.e., 6.7% on each of day 2, days 3-7 and days 8-41).

Within the community (births outside of health facilities), barriers currently inhibiting timely vaccination coverage include suboptimal qualified health worker attendance and/or travel distance from a health facility where birth dose vaccines are kept [15-17]. To capture these potential latencies, we assumed that 30% of baseline coverage in the community was timely (<24 hours), 40% delivered on day 2, and 15% on each of the days 3-7 and days 8–41-time strata.

Appendix 3: Additional details on vaccination costing

Vaccine Supply Chain Costs

A supply chain cost component was used to account for the costs associated with transporting vaccines from a national store to a health facility, and storage of vaccines in a cold chain. Economic costs were taken from a review by Portnoy and colleagues, which estimated the non-commodity costs of introducing a new vaccine into a routine immunization program [18]. Costs were available for 78 (98%) modelled LMICs and assumed constant for both vial and MAP presentation of vaccine.

Source costs were presented in 2018 US$ and modelled in 2020 US$ (CPI adjusted). Impacts of alternate assumptions for MAPs were quantified in a one-way sensitivity analysis (Main Text, Fig 3).

Table E: Modelled per dose supply chain costs for each WHO region (2020 US$; population-weighted averages)

	AFRO	AMRO	EMRO	EURO	SEARO	WPRO	All LMICs
Supply Chain Cost (95% CI)	$2.33 ($0.83, $5.33)	$2.67 ($0.82, $6.77)	$2.46 ($0.89, $5.49)	$3.74 ($1.28, $8.81)	$1.23 ($0.31, $3.41)	$2.11 ($0.57, $5.65)	$1.96 ($0.58, $5.03)

Vaccine Commodity Costs

Within the model, per dose commodity costs considered three individual components: vaccines, needle and syringe, disposal boxes; plus, an allowance for wastage where required. Across all modelled LMICs, commodity costs were assumed equal.

- **Vaccines:** Baseline birth dose coverage assumed a combination of 10-dose (MDV) and single-dose (SDV) vials were being used for vaccinations. Modelled costs were the average, and uncertainty the range, of available UNICEF-Supply and Demand (UNICEF-SD) price points for pediatric presentations in 2020 [19]. A procurement ratio of 3 MDV: 1 SDV was used in analysis, guided by UNICEF purchasing data [20]. For MAP presentations, vaccine costs of US$1.65, US$3.30 and US$5.00 were investigated as part of analysis.

- **Vaccine Wastage:** Vaccine wastage rates were estimated using the WHO Vaccine Wastage Calculator tool [21]. A wastage rate of 17.5% (range: 10%, 25%) was applied for MDV vaccines and 4% (range: 3%, 5%) for SDVs. Cost-effectiveness outcomes for MAP vaccines included a 4% wastage allowance, consistent with other single dose presentations.

- **Needle and Syringe:** 0.5mL auto-disable needle and syringes are needed for vial presentations and included in the cost estimates. Modelled costs were the average, and uncertainty the range, of UNICEF-SD price points for devices in 2020 [22]. We assumed a 10% wastage rate for needles and syringes, consistent with UNICEF and WHO assumptions for procurement. A needle and syringe component were not needed for MAP presentations.

- **Disposal Box:** 5L disposal boxes were included in the per dose cost for vial vaccines. Modelled costs were the average, and uncertainty the range, of UNICEF-SD price points for devices in 2020 [22]. Per dose costs were calculated assuming each disposal box could hold

100 0.5mL auto-disable needle and syringe devices and included allowance for needle and syringe wastage. A disposal box component was assumed as not required for disposal of MAPs.

Table F: Modelled per dose vaccine commodity costs (2020 USD); assumed fixed for each modelled setting.

	Per Dose vaccine cost (range)	Vaccine Wastage*, % (range)	Per dose 0.5mL auto disable needle and syringe cost (range)	0.5mL auto disable needle and syringe wastage*, % (range)	Per dose disposal box cost (range)	Per dose total commodity cost (range)
Multi Dose Vial (MDV)	$0.25 ($0.24, $0.25)	17.5 (10, 25)	$0.041 ($0.031, $0.066)	10%	$0.0062 ($0.0045, $0.0093)	$0.34 ($0.31, $0.41)
Single Dose Vial (SDV)	$0.55 ($0.49, $0.60)	4 (3, 5)	$0.041 ($0.031, $0.066)	10%	$0.0062 ($0.0045, $0.0093)	$0.62 ($0.54, $0.71)
3 MDV: 1 SDV (baseline) weighted estimate	---	----	---	---	---	$0.41 ($0.36, $0.48)
Microarray Patch (MAP)	$1.65/$3.30/$5.00	4	---	---	---	$1.72/$3.44/$5.21

* Per dose wastage cost for vaccines and 0.5mL auto disable needle and syringes calculated as: *unit cost*(1/1-wastage (%))*

Human Resource (Vaccine Administration Time) Costs

Human resource costs captured the health worker time required to deliver a hepatitis B birth dose vaccine. We limited time to the administration of a birth dose vaccination from a given vaccine presentation (SDV, MDV, MAP) and excluded time-cost associated with provision of other post-birth services and other vaccine program activities. A PATH time-in-motion analysis was used to estimate vaccination delivery times, weighted proportional to MDV and SDV usage where relevant [23]. Delivery times for a CPAD within the PATH analysis were used as a proxy for MAPs, consistent with application times in a childhood MAP vaccination study [24]. However, target product profiles for MAPs indicate this time could be much longer and remain acceptable for use [25], hence the impact was evaluated in one-way sensitivity analysis (Main Text, Fig 3).

Valuation of time was linked to per capita GDP, using multipliers from an econometric analysis by Serje and colleagues according to World Bank Income Classification status [5]. Health workers were assumed to work 37.5-hour weeks for 48 weeks per year and that doctors, nurses and midwives would vaccinate births with an equal probability; however, costs were weighted proportionally to the number of each cadre within a LMIC [26, 27]. Valuation of trained lay-health workers to administer MAPs to births in the community used the "other health workers" multiplier as a proxy.

Regional analysis used population weighted (2020 births) averages across relevant subsets of LMICs.

Table G: Vaccine administration time for each modelled modality. Values approximated from a PATH time and motion analysis [23]

Vaccine Delivery Modality	Administration Time (seconds)	Range
10-Dose Vial (MDV)	15.2	8-35
Single Dose Vial (SDV)	19.3	10-40
MAP (using CPAD as proxy)	7.6	5-20

Table H: Modelled per dose human resource costs (2020 USD) for each WHO region; uncertainty represents lower and upper bound vaccine administration time estimates.

	AFRO	AMRO	EMRO	EURO	SEARO	WPRO	All LMICs
Cold Chain, QHW	$0.02 (0.01-0.05)	$0.05 (0.02-0.10)	$0.03 (0.02-0.07)	$0.04 (0.02-0.09)	$0.03 (0.01-0.06)	$0.06 (0.03-0.13)	$0.04 (0.02-0.08)
MAPs, QHW	$0.01 (0.007-0.03)	$0.02 (0.01-0.06)	$0.01 (0.009-0.04)	$0.02 (0.01-0.05)	$0.01 (0.008-0.03)	$0.03 (0.02-0.07)	$0.02 (0.01-0.05)
MAPs, LHW	$0.006 (0.004-0.02)	$0.01 (0.008-0.03)	$0.007 (0.005-0.02)	$0.01 (0.007-0.03)	$0.006 (0.004-0.02)	$0.01 (0.009-0.04)	$0.01 (0.006-0.03)

QHW: Qualified Health Worker, LHW: Trained Lay-Health Worker, CTC: Controlled Temperature Chain, MAP: Microarray Patch

Outreach Costs

Outreach aimed to capture costs associated with providing a birth dose vaccination in the community, including transport costs and travel time. Costs were taken from an analysis by Nayagam and colleagues [4]; however, costs in the South Asia GBD Super Region were deemed unrealistic

($32 per dose, 2020 USD). For LMICs within this region, a population weighted (2020 births) average of outreach costs elsewhere was used *in lieu*.

As the study only provided point estimate costs, uncertainty was modelled as a uniform ±5% from point estimates.

Table I: Modelled per dose outreach costs to reach a birth within the community (2020 USD) for each WHO region.

	AFRO	AMRO	EMRO	EURO	SEARO	WPRO	All LMICs
Outreach Cost	$3.59	$0.02	$0.49	$2.94	$1.55	$0.64	$1.18

Appendix 4: Additional figures and tables

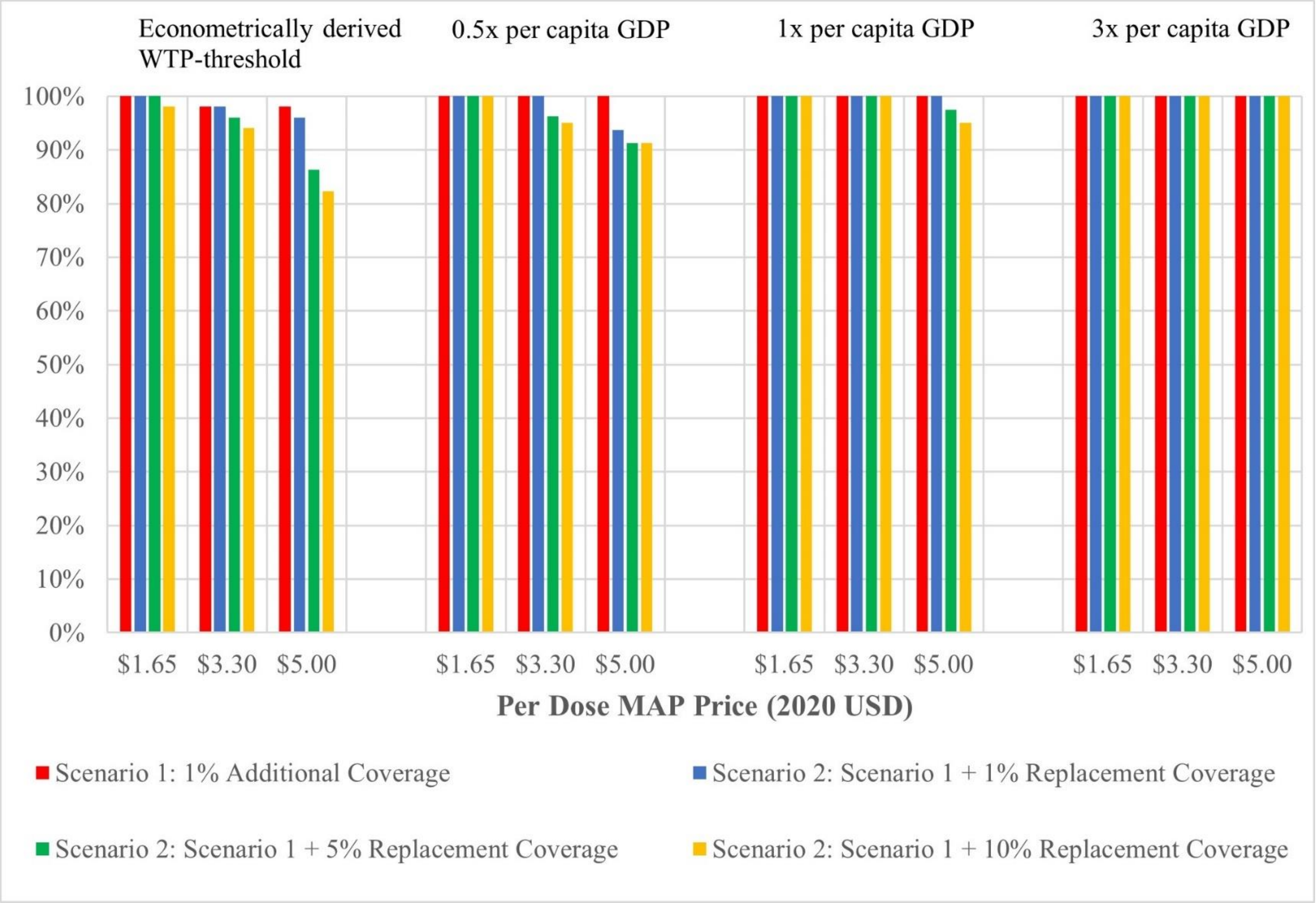

Fig B: Cost-effectiveness of MAPs against willingness-to-pay (WTP) thresholds, using median ICERs across three investigated MAP price points for 80 LMICs.

Note: Published estimates of WTP per DALY averted were only available for 51/80 (64%) of modelled LMICs [28].

Appendix 5: Supplemental Analyses

Sensitivity analysis: larger coverage gains due to MAPs

Incremental gains in additional coverage only (scenario 1) were associated with greater health benefits (Table L); however, did not impact cost-effectiveness of MAPs (i.e., ICERs did not change).

Cost-effectiveness of replacement coverage (scenario 2) was enhanced with higher levels of additional coverage, but remained less cost-effective (i.e., higher ICERs) compared to additional coverage only (scenario 1).

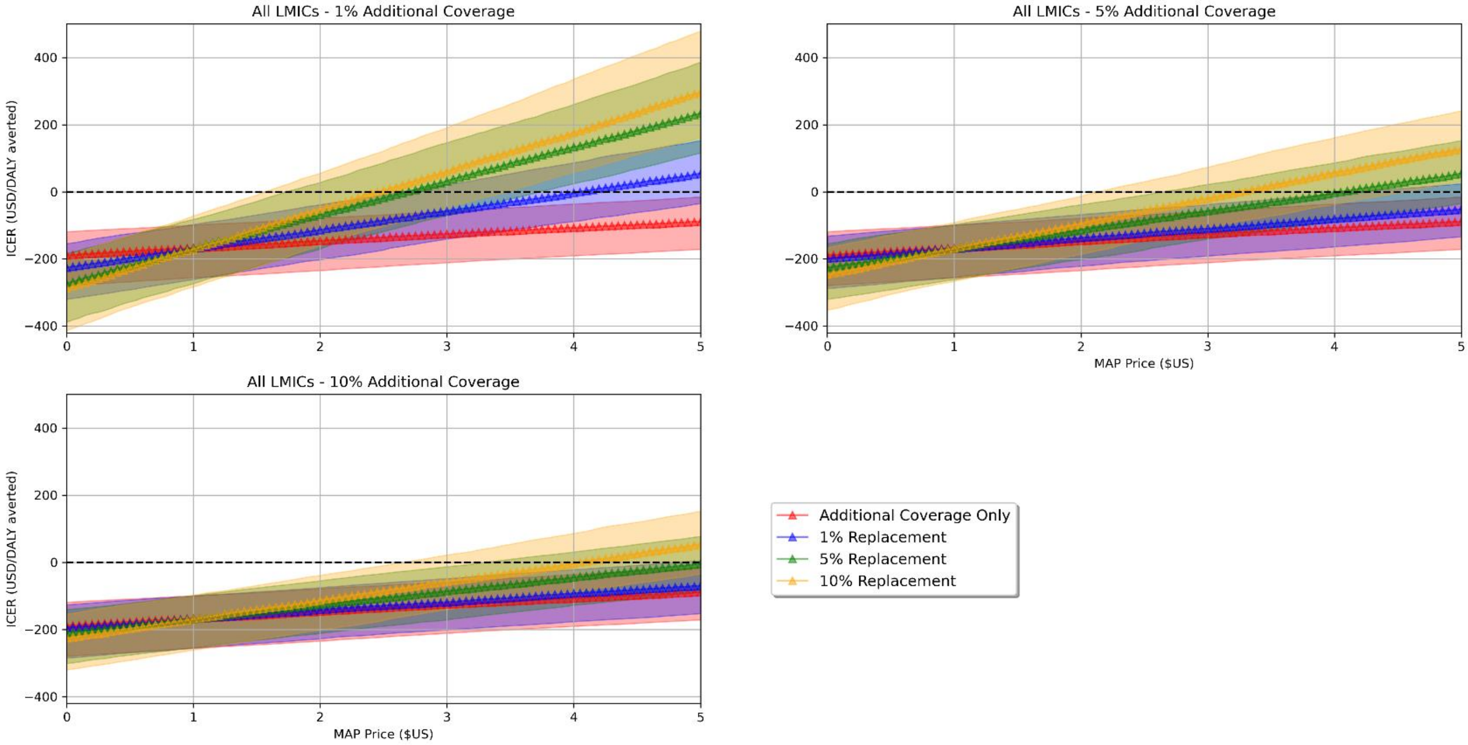

Fig C: Outcomes of assumptions on incremental coverage gains from MAPs within the model. Shaded region represents Interquartile Range (IQR) of 1000 model simulations.

Table L: Outcomes of sensitivity analysis where MAPs provide larger coverage gains.

	MAPs Administered	DALYs Averted (vs baseline)	Disease Management Costs ('000 USD)	Vaccination Costs (excluding MAP commodity costs; '000 USD)	ICER, USD per DALY averted (MAP Procurement Price = US$1.65 per dose)	ICER, USD per DALY averted (MAP Procurement Price = US$3.30 per dose)	ICER, USD per DALY averted (MAP Procurement Price = US$5.00 per dose)
**All LMICs (80 countries, 80.1 million births)**							
Baseline (MDV+SDV)	--	1,538,536 (1,224,957; 1,868,764)	399,541 (287,497; 541,512)	163,807 (136,555; 203,261)	--	--	--
**1% Additional Coverage**							
+1% additional coverage with MAPs	49,162 (43,425; 55,272)	2,468 (1,936: 3,116)	398,795 (287,021; 540,606)	163,955 (136,714; 203,425)	-154.44 (-242.33; -86.88)	-119.64 (-204.17; -52.48)	-88.65 (-171.22; -15.44)
+1% additional and 1% replacement coverage with MAPs	194,369 (184,273; 204,183)	3,476 (2,785: 4,398)	398,507 (286,803; 540,317)	163,966 (136,808; 203,421)	-132.86 (-222.07; -59.75)	-41.04 (-126.66; 40.51)	54.40 (-34.88; 152.73)
+1% additional and 5% replacement coverage with MAPs	773,426 (746,364; 799,418)	7,552 (5,952: 9,643)	397,357 (286,139; 539,162)	163,647 (136,855; 202,958)	-101.92 (-205.27; -9.78)	64.17 (-41.07; 179.19)	233.64 (115.71; 386.49)
+1% additional and 10% replacement coverage with MAPs	1,498,144 (1,446,550; 1,546,363)	12,640 (9,818: 16,240)	395,919 (285,373; 537,718)	163,427 (136,590; 202,456)	-92.77 (-205.03; 11.27)	98.03 (-16.26; 233.87)	296.78 (161.09; 478.89)
**5% Additional Coverage**							
+5% additional coverage with MAPs	245,808 (217,126; 264,490)	12,431 (9,678; 15,582)	395,811 (285,266; 537,258)	164,646 (137,233; 204,062)	-154.44 (-242.33; -86.88)	-119.64 (-204.17; -52.48)	-88.65 (-171.22; -15.44)
+5% additional and 1% replacement coverage with MAPs	391,625 (358,144; 425,403)	13,332 (10,626; 16,778)	395,525 (285,138; 536,767)	164,591 (137,295; 203,948)	-148.97 (-232.59, -78.75)	-101.56 (-182.51, -28.27)	-52.63 (-133.98, 24.32)
+5% additional and 5% replacement coverage with MAPs	971,847 (921,367; 1,020,915)	17,381 (13,924; 21,990)	394,373 (284,535; 535,472)	164,423 (137,379; 203,503)	-132.86 (-222.07; -59.75)	-41.04 (-126.66; 40.51)	54.40 (-34.88; 152.73)
+5% additional coverage and 10% replacement with MAPs	1,694,224 (1,625,501; 1,759,731)	22,517 (18,002; 28,408)	392,935 (283,705; 534,092)	164,197 (137,319; 202,924)			
**10% Additional Coverage**							
+10% additional coverage with MAPs	491,616 (434,251; 552,723)	24,681 (19,357; 31,163)	392,105 (283,201; 532,972)	165,446 (138,162; 204,593)	-154.44 (-242.33; -86.88)	-119.64 (-204.17; -52.48)	-88.65 (-171.22; -15.44)
+10% additional and 1% replacement coverage with MAPs	637,162 (574,247; 701,797)	25,681 (20,356; 32,324)	391,794 (283,013; 532,578)	165,363 (138,173; 204,479)	-150.76 (-237.16, -84.18)	-110.59 (-193.71, -39.62)	-70.12 (-152.20, 4.37)

+10% additional and 5% replacement coverage with MAPs	1,218,070 (1,127,299; 1,298,061)	29,680 (23,774; 37,520)	390,644 (282,288; 530,614)	165,367 (138,228; 204,213)	-140.87 (-225.45; -69.69)	-73.39 (-157.11, 0.02)	-3.91 (-88.93; 77.60)
+10% additional and 10% replacement coverage with MAPs	1,943,693 (1,842,733; 2,041,830)	37,763 (27,847, 43,981)	389,206 (281,568; 529,147)	165,011 (138,364, 203,668)	-132.86 (-222.07; -59.75)	-41.04 (-126.66; 40.51)	54.40 (-34.88; 152.73)

Uncertainty parenthesized as the Interquartile Range (IQR) of 1000 model iterations. Costs presented in 2020 USD. Negative ICERs indicative of cost-savings (i.e., health benefits achieved at a lower overall cost compared to baseline expenditure).

Abbreviations: MAP: Microarray Patch, MDV: Multiple Dose Vial, SDV: Single Dose Vial.

Sensitivity analysis: MAPs do not create new coverage, but are used to replace existing needle and syringe coverage (and hence improve timing of delivery only)

Cost-effectiveness of using MAPs to replace existing coverage only (Supplemental Figure 3, right panel) was equal across all analyzed increments (1%, 5% and 10%). While less cost-effective than additional coverage from MAPs (i.e., higher ICERs for a given MAP price), this sensitivity analysis indicates use of MAPs to only achieve gains in birth dose timeliness may present some value.

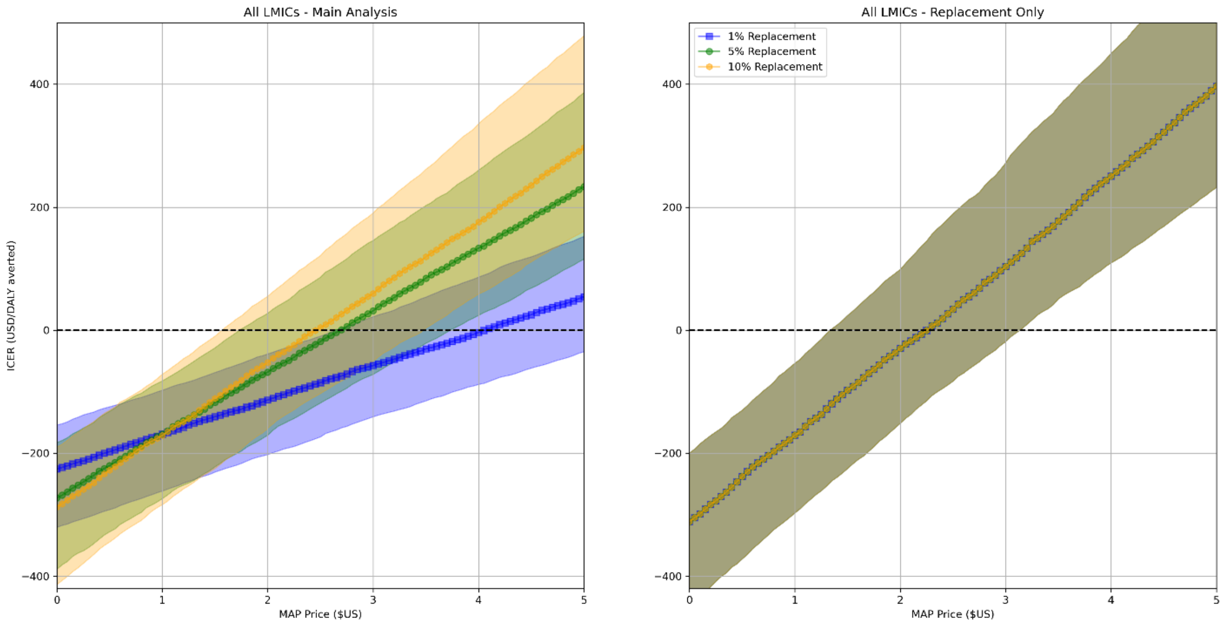

Fig D: Comparative outcomes of MAPs when replacement coverage is paired with additional coverage (left) and when modelled in isolation (right). Shaded region represents Interquartile Range (IQR) of 1000 model simulations.

Sensitivity analysis: When MAPs replace existing coverage it always results in day 1 coverage, rather than a left shift in the timing of delivery

Instead of a left shift in timing distribution of vaccines delivered by qualified health workers, this sensitivity analysis explored a best-case scenario: MAPs enable all delayed vaccinations to occur on the day of birth (i.e., timely).

Subsequent gains in vaccine effectiveness mean that cost-effectiveness of MAPs is enhanced, relative to baseline assumptions.

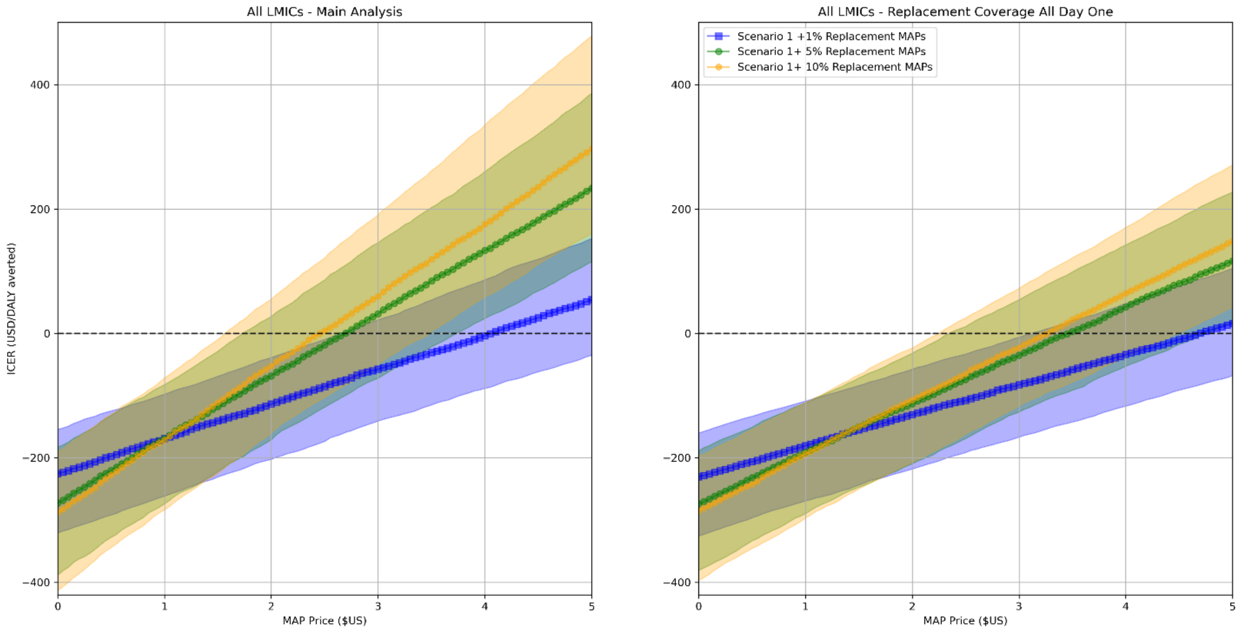

Fig E: Comparative outcomes of MAPs when timing gains from maps are left shifted one time-strata (left) or if all replacement coverage is shifted to the day of birth (right) within the model. Shaded region represents Interquartile Range (IQR) of 1000 model simulations.

Sensitivity analysis: MAPs also provide additional, new coverage in facilities (rather than just additional coverage in the community).

For this supplemental analysis, we assumed that additional coverage from MAPs in facilities was half of that in the community (i.e. 0.5% additional coverage). However, as the majority of births remain in health facilities and vaccination does not incur an outreach cost, even constrained use in this setting improves cost-effectiveness.

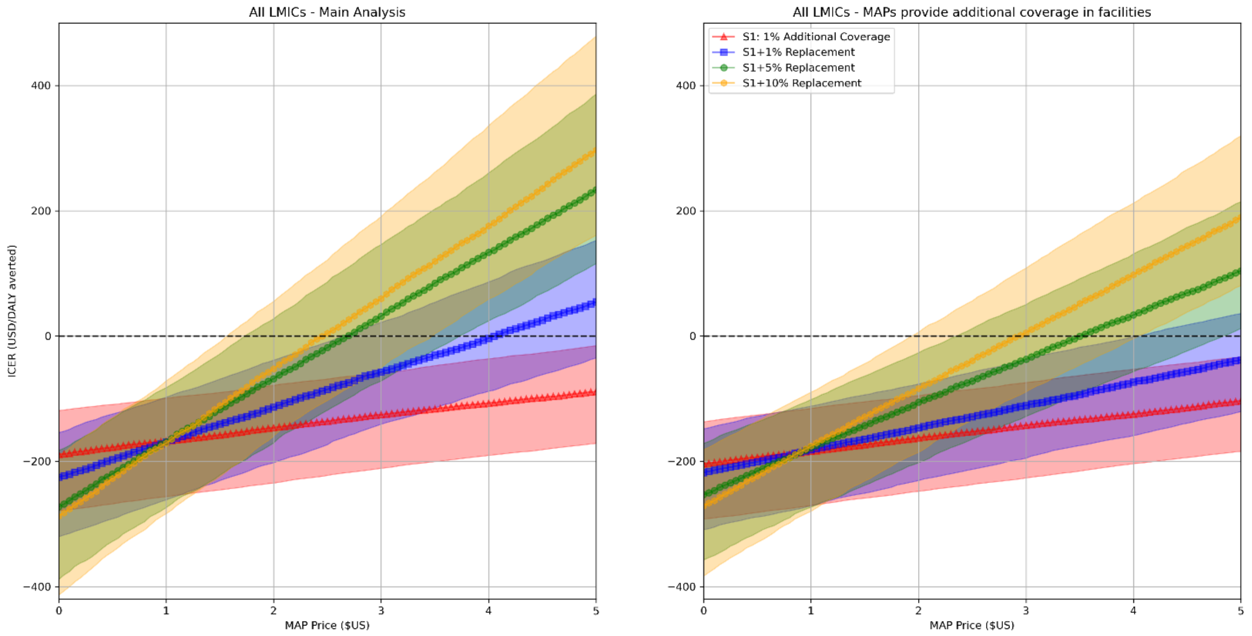

Fig F: Comparative outcomes of MAPs when additional coverage is limited to community births only (left) or if additional coverage can also occur for facility births (right) within the model. Shaded region represents Interquartile Range (IQR) of 1000 model simulations.

Appendix 6: Sensitivity Analysis – cost-effectiveness of MAPs if implemented with a baseline CTC approach

The hepatitis B birth dose is deemed a CTC priority vaccine by the WHO CTC Working Group [29]. In this sensitivity analysis, we investigated how a theoretical baseline scenario where the CTC approach was already being used would impact the cost-effectiveness of MAPs to deliver the birth dose.

We assumed that all vial vaccines used under a CTC approach would be single-dose, and that each vial would be fitted with a combined vaccine vial monitor (VVM) and threshold temperature indicator (TTI). As vaccines were in vials, administration remained a task for qualified health workers only (i.e., required a needle and syringe). Consistent with previous CTC hepatitis B birth dose modelling studies [30, 31], at baseline, CTC provided an additional 5% timely (day 1) coverage of births in facilities and 10% timely coverage of births in the community. Additionally, CTC improves timeliness of 5% of facility and 10% of community vaccinations by replacing existing cold chain coverage, modelled as a left shift (i.e., days 8-41 to days 3-7, days 3-7 to day 2, and day 2 to day 1) in vaccine timing.

Results from this analysis suggest introduction of MAPs under a baseline scenario with a CTC approach would have negligible impact on their cost-effectiveness, as compared to a baseline scenario entirely reliant upon the cold chain.

Table M: Per dose component costs of vial vaccines delivered under a CTC approach, presented in $US 2020.

	**All LMICs**
Supply Chain Cost [18]	$1.96 ($0.58, $5.03)
Vaccine Cost (inc. needle and syringe, disposal and wastage) [19, 22]	$0.65 ($0.57, $0.75)
Temperature Threshold Indicator (TTI) [32]	$0.035 ($0.03, $0.04)
Delivery Cost [5, 23, 26, 27]	$0.04 ($0.02, $0.09)
Outreach Cost (community births) [4]	$1.18

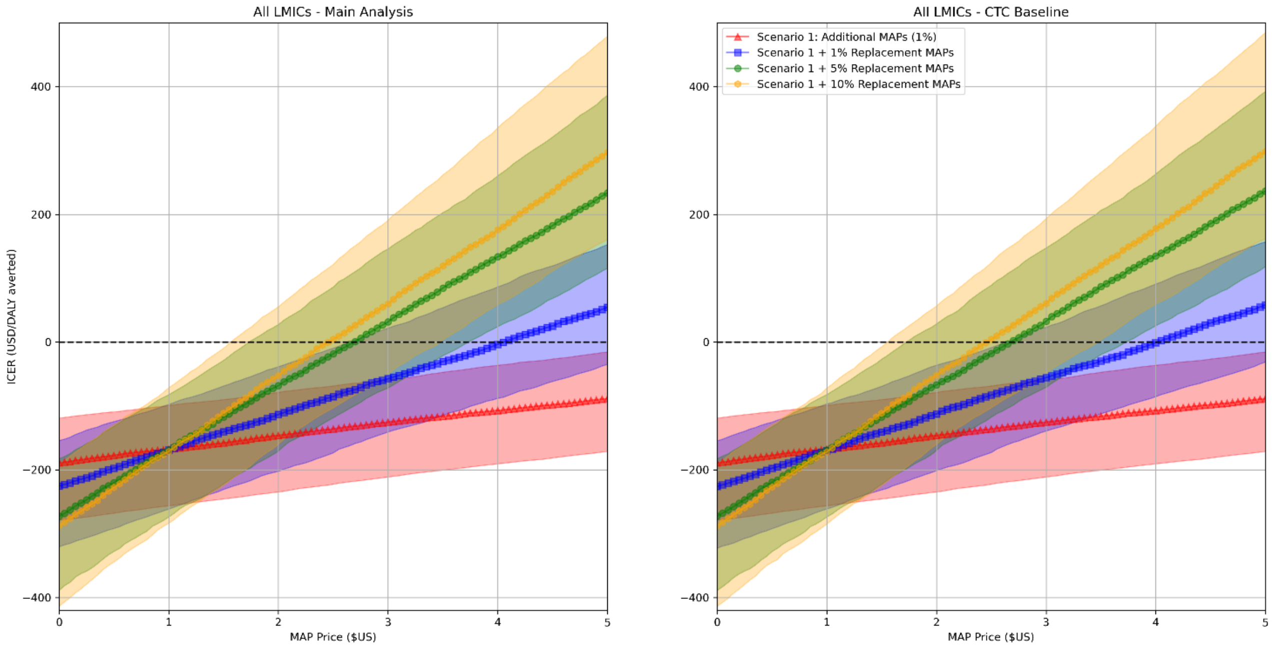

Fig G: Comparative outcomes of MAPs when comparing a cold chain baseline (left) with a hypothetical future controlled temperature chain (CTC; right) baseline within the model. Shaded region represents IQR of 1000 model simulations.

Table N: Sensitivity analysis of MAP cost-effectiveness for delivering the hepatitis B birth dose in 77 LMICs under cold chain and cold chain plus CTC baseline assumptions. Costs in US$ 2020, uncertainity in parenthesis represents IQR of 1000 model iterations.

	**MAPs Administered**	**DALYs Averted (for MAPs additional DALYs averted vs baseline)**	**Disease Management Costs ('000 USD)**	**Vaccination Costs (excluding MAP commodity costs; '000 USD)**	**ICER, USD per DALY averted (MAP Procurement Price = US$1.65 per dose)**	**ICER, USD per DALY averted (MAP Procurement Price = US$3.30 per dose)**	**ICER, USD per DALY averted (MAP Procurement Price = US$5.00 per dose)**
**Cold Chain Only (80 countries, 80.1 million births)**							
Baseline (MDV+SDV)	---	1,538,536 (1,224,957; 1,868,764)	399,541 (287,497; 541,512)	163,807 (136,555; 203,261)	---	---	---
+1% additional coverage with MAPs	49,162 (43,425; 55,272)	2,468 (1,936: 3,116)	398,795 (287,021; 540,606)	163,955 (136,714; 203,425)	-154.44 (-242.33; -86.88)	-119.64 (-204.17; -52.48)	-88.65 (-171.22; -15.44)
+1% additional and 1% replacement coverage with MAPs	194,369 (184,273; 204,183)	3,476 (2,785: 4,398)	398,507 (286,803; 540,317)	163,966 (136,808; 203,421)	-132.86 (-222.07; -59.75)	-41.04 (-126.66; 40.51)	54.40 (-34.88; 152.73)
+1% additional and 5% replacement coverage with MAPs	773,426 (746,364; 799,418)	7,552 (5,952: 9,643)	397,357 (286,139; 539,162)	163,647 (136,855; 202,958)	-101.92 (-205.27; -9.78)	64.17 (-41.07; 179.19)	233.64 (115.71; 386.49)
+1% additional and 10% replacement coverage with MAPs	1,498,144 (1,446,550; 1,546,363)	12,640 (9,818: 16,240)	395,919 (285,373; 537,718)	163,427 (136,590; 202,456)	-92.77 (-205.03; 11.27)	98.03 (-16.26; 233.87)	296.78 (161.09; 478.89)
**Cold Chain plus CTC**							
Baseline (MDV+SDV)	---	1,459,002 (1,161,725; 1,771,574)	378,708 (272,971; 514,234)	168,350 (141,631; 207,187)	---	---	---
+1% additional coverage with MAPs	44,245 (39,083; 49,745)	2,221 (1,742; 2,805)	378,056 (272,587; 513,343)	168,462 (141,792, 207,369)	-154.44 (-242.33; -86.88)	-119.64 (-204.17; -52.48)	-88.65 (-171.22; -15.44)
+1% additional and 1% replacement coverage with MAPs	180,029 (171,063, 188,786)	3,164 (2,540; 4,005)	377,718 (272,406; 513,086)	168,457 (141,846; 207,318)	-132.82 (-222.16; -59.04)	-39.45 (-123.12, 44.46)	57.97 (-30.94; 156.98)
+1% additional and 5% replacement coverage with MAPs	721,394 (697,457; 744,803)	6,999 (5,508; 8,932)	376,507 (271,866; 521,058)	168,572 (141,896; 206,988)	-101.61 (-205.22, -9.07)	65.83 (-39.44; 182.88)	236.39 (118.49; 392.54)
+1% additional and 10% replacement coverage with MAPs	1,399,969 (1.352,838; 1,443,121)	11,760 (9,110; 15,140)	375,160 (271,085; 510,746)	168,455 (141,806; 206,588)	-92.42 (-204.51, 13.67)	97.92 (-15.92, 236.72)	297.95 (161.62, 484.54)

Negative ICER (Incremental Cost Effectiveness Ratio) reflective of dominance (cost-savings over the cohort lifetime). CTC = Controlled Temperature Chain, MDV = 10-dose vial, SDV = Single Dose Vial, MAP = Microarray Patch

References:

1. WHO-UNICEF. Estimates of childhood immunization coverage [cited 2021 Feburary 15]. Available from: https://apps.who.int/immunization_monitoring/globalsummary/timeseries/tswucoveragehepb_bd.html .
2. United Nations DoE, Social Affairs PD. World Population Prospects 2019, Online Edition. Rev. 1. 2019.
3. Ott JJ, Stevens GA, Wiersma ST. The risk of perinatal hepatitis B virus transmission: hepatitis B e antigen (HBeAg) prevalence estimates for all world regions. BMC Infect Dis. 2012;12:131. Epub 2012/06/12. doi: 10.1186/1471-2334-12-131. PubMed PMID: 22682147; PubMed Central PMCID: PMCPMC3478174.
4. Nayagam S, Thursz M, Sicuri E, Conteh L, Wiktor S, Low-Beer D, et al. Requirements for global elimination of hepatitis B: a modelling study. Lancet Infect Dis. 2016;16(12):1399-408. Epub 2016/09/18. doi: 10.1016/S1473-3099(16)30204-3. PubMed PMID: 27638356.
5. Serje J, Bertram MY, Brindley C, Lauer JA. Global health worker salary estimates: an econometric analysis of global earnings data. Cost Effectiveness and Resource Allocation. 2018;16(1):1-9.
6. World Health Organization. WHO Regional Offices [15 Feburary, 2021)]. Available from: https://www.who.int/about/who-we-are/regional-offices.
7. IHME. Global Health Data Exchange - Countries [15 Feburary, 2021)]. Available from: http://ghdx.healthdata.org/countries.
8. The World Bank. The World by Income and Region [15 Feburary, 2021)]. Available from: https://datatopics.worldbank.org/world-development-indicators/the-world-by-income-and-region.html.
9. Breakwell L, Tevi-Benissan C, Childs L, Mihigo R, Tohme R. The status of hepatitis B control in the African region. Pan Afr Med J. 2017;27(Suppl 3):17. Epub 2018/01/04. doi: 10.11604/pamj.supp.2017.27.3.11981. PubMed PMID: 29296152; PubMed Central PMCID: PMCPMC5745934.
10. Nayagam S, Shimakawa Y, Lemoine M. Mother-to-child transmission of hepatitis B: what more needs to be done to eliminate it around the world? Journal of viral hepatitis. 2020;27(4):342-9.
11. Hu Y, Chen Y, Wang Y, Liang H. Hepatitis B Vaccination among 1999(-)2017 Birth Cohorts in Zhejiang Province: The Determinants Associated with Infant Coverage. Int J Environ Res Public Health. 2018;15(12). Epub 2018/12/24. doi: 10.3390/ijerph15122915. PubMed PMID: 30572681; PubMed Central PMCID: PMCPMC6313558.
12. Olakunde BO, Adeyinka DA, Olakunde OA, Ogundipe T, Oladunni F, Ezeanolue EE. The coverage of hepatitis B birth dose vaccination in Nigeria: Does the place of delivery matter? Transactions of The Royal Society of Tropical Medicine and Hygiene. 2021.
13. Breakwell L, Anga J, Dadari I, Sadr-Azodi N, Ogaoga D, Patel M. Evaluation of storing hepatitis B vaccine outside the cold chain in the Solomon Islands: Identifying opportunities and barriers to implementation. Vaccine. 2017;35(21):2770-4. PubMed PMID: 615431212.
14. Patel MK, Capeding RZ, Ducusin JU, de Quiroz Castro M, Garcia LC, Hennessey K. Findings from a hepatitis B birth dose assessment in health facilities in the Philippines: opportunities to engage the private sector. Vaccine. 2014;32(39):5140-4. Epub 2013/12/24. doi: 10.1016/j.vaccine.2013.11.097. PubMed PMID: 24361121; PubMed Central PMCID: PMCPMC4663669.
15. Garces A, McClure EM, Chomba E, Patel A, Pasha O, Tshefu A, et al. Home birth attendants in low income countries: who are they and what do they do? BMC Pregnancy Childbirth. 2012;12:34. Epub 2012/05/16. doi: 10.1186/1471-2393-12-34. PubMed PMID: 22583622; PubMed Central PMCID: PMCPMC3493311.
16. Creati M, Saleh A, Ruff TA, Stewart T, Otto B, Sutanto A, et al. Implementing the birth dose of hepatitis B vaccine in rural Indonesia. Vaccine. 2007;25(32):5985-93. Epub 2007/07/03. doi: 10.1016/j.vaccine.2007.05.055. PubMed PMID: 17604881.

17. Murakami H, Van Cuong N, Huynh L, Hipgrave DB. Implementation of and costs associated with providing a birth-dose of hepatitis B vaccine in Viet Nam. Vaccine. 2008;26(11):1411-9. Epub 2008/02/12. doi: 10.1016/j.vaccine.2008.01.002. PubMed PMID: 18262312.
18. Portnoy A, Vaughan K, Clarke-Deelder E, Suharlim C, Resch SC, Brenzel L, et al. Producing standardized country-level immunization delivery unit cost estimates. PharmacoEconomics. 2020;38(9):995-1005.
19. UNICEF. HepB vaccine price data. Available from: https://www.unicef.org/supply/media/271/file/HepB%20vaccine%20price%20data.pdf.
20. UNICEF. UNICEF Pricing Data [cited 2021 Feburary 15]. Available from: https://www.unicef.org/supply/pricing-data.
21. WHO. Vaccine Wastage Rates Calculator [May 17, 2021)]. Available from: https://www.who.int/publications/m/item/vaccine-wastage-rates-calculator.
22. UNICEF. Syringes and Safety Boxes Price Data [May 6, 2021)]. Available from: https://www.unicef.org/supply/media/2881/file/%20syringes%20-safety-boxes-price-data-April2020.pdf.
23. PATH. Pentavalent Vaccine in the Uniject™ Injection System: A Time and Motion Study [06 May, 2021)]. Available from: https://path.azureedge.net/media/documents/TS_pentavalent_vac.pdf.
24. Guillermet E, Alfa DA, Subedi M, Demolis R, Giersing B, Jaillard P. End-user acceptability study of the nanopatch™; a microarray patch (MAP) for child immunization in low and middle-income countries. Vaccine. 2019;37(32):4435-43.
25. World Heath Organization and The United Children's Fund. Measles-rubella microarray patch (MR–MAP) target product profile [15 May 2021]. Available from: https://www.who.int/immunization/research/ppc-tpp/WHO_MR_MAP_TPP.pdf.
26. World Health Organization. Global Health Observatory: Medical doctors (per 10 000 population) [cited 2021 Feburary 15]. Available from: https://www.who.int/data/gho/data/indicators/indicator-details/GHO/medical-doctors-(per-10-000-population).
27. World Health Organization. Global Health Observatory: Nursing and Midwifery personnel (per 10 000 population) [cited 2021 Feburary 15]. Available from: https://www.who.int/data/gho/data/indicators/indicator-details/GHO/nursing-and-midwifery-personnel-(per-10-000-population).
28. Ochalek J, Lomas J, Claxton K. Estimating health opportunity costs in low-income and middle-income countries: a novel approach and evidence from cross-country data. BMJ Glob Health. 2018;3(6):e000964. Epub 2018/11/30. doi: 10.1136/bmjgh-2018-000964. PubMed PMID: 30483412; PubMed Central PMCID: PMCPMC6231096.
29. World Health Organization. Controlled temperature chain: strategic roadmap for priority vaccines 2017-2020. World Health Organization, 2017.
30. Scott N, Palmer A, Morgan C, Lesi O, Spearman CW, Sonderup M, et al. Cost-effectiveness of the controlled temperature chain for the hepatitis B virus birth dose vaccine in various global settings: a modelling study. The Lancet Global Health. 2018;6(6):e659-e67. PubMed PMID: 2000753269.
31. Seaman CP, Morgan C, Howell J, Xiao Y, Spearman CW, Sonderup M, et al. Use of controlled temperature chain and compact prefilled auto-disable devices to reach 2030 hepatitis B birth dose vaccination targets in LMICs: a modelling and cost-optimisation study. The Lancet Global Health. 2020;8(7):e931-e41.
32. Vaccine Innovation Prioritisation Strategy (VIPS). VIPS PHASE 2 TECHNICAL NOTE Combined Vaccine Vial Monitor and Threshold Indicator [May 21, 2021)]. Available from: https://www.gavi.org/sites/default/files/about/market-shaping/Phase-II/3_VIPS%20Phase%20II%20Technical%20Note%20Combined%20Vaccine%20Vial%20Monitor%20%28VVM%29%20and%20Threshold%20Indicators%20%28TI%29.pdf.
